# FBXL6 promotes bladder cancer progression by stabilizing ENO1 through K63-linked ubiquitination

**DOI:** 10.1038/s41420-026-03130-x

**Published:** 2026-05-06

**Authors:** Ruoyu Huang, Jingtian Yu, Renran Bai, Wenyu Jiang, Fenfang Zhou, Tongzu Liu, Xinghuan Wang

**Affiliations:** 1 Department of Urology, Zhongnan Hospital of Wuhan University, Wuhan, China; 2 Department of Radiology, Zhongnan Hospital of Wuhan University, Wuhan, China; 3 Hubei Key Laboratory of Urological Diseases, Wuhan University, Wuhan, China

**Keywords:** Bladder cancer, Cancer metabolism

## Abstract

The role of ubiquitin in post-translational modifications is important for tumor progression, but how these mechanisms regulate bladder cancer (BLCA) is not completely known. The study demonstrated that FBXL6, a member of the F-box protein family, could drive oncogenesis in BLCA, as shown by integrative bioinformatic analysis and clinical sample validation. Experiments demonstrated that FBXL6 speeds up the in vitro growth and migration of BLCA cells and contributes to tumor development and metastasis in vivo. Mechanistically, transcriptomic and metabolic studies indicate that FBXL6 regulates the glycolytic pathway. Although FBXL6 knockdown has minimal impact on the mRNA levels of the key glycolytic enzyme ENO1, FBXL6 knockdown does alter ENO1 protein levels, suggesting post-translational regulation. Co-immunoprecipitation and GST pull-down assays validated the interaction between FBXL6 and ENO1, confirming that the LRR domain of FBXL6 and the C-terminal region of ENO1 are essential for their binding. Additionally, ubiquitination assays indicated that FBXL6 promotes K63-linked polyubiquitination of ENO1, which stabilizes it. Bringing back ENO1 expression partially offset the consequences of FBXL6 knockdown on proliferation and migration. In summary, our findings propose a new model where FBXL6 promotes BLCA progression by stabilizing ENO1 through K63 linkage, emphasizing its potential as a target for BLCA therapy.

## Introduction

Bladder cancer (BLCA) ranks among the most frequently diagnosed malignancies of the urinary tract, accounting for an estimated 573,000 new diagnoses and 213,000 deaths reported globally in 2020 [1]. Despite advances in surgical and immunotherapeutic strategies, the prognosis for advanced or recurrent BLCA remains poor, largely due to the high metastatic potential and metabolic plasticity of tumor cells [2, 3]. Reprogramming of cellular metabolism, particularly the enhancement of aerobic glycolysis [4, 5], has emerged as a hallmark of cancer progression, driving tumor cell proliferation [6], migration [7], and resistance to therapy [8]. However, the upstream regulatory mechanisms governing glycolytic reprogramming in BLCA remain incompletely understood.

The ubiquitin-proteasome system, particularly E3 ubiquitin ligases, plays a pivotal role in regulating protein stability and function, and has been increasingly implicated in tumorigenesis [9–13]. Among the various E3 ligases, F-box proteins are core components of the SCF (SKP1/CUL1/F-box) ubiquitin ligase complex, functioning as substrate recognition modules [14–18]. F-box and leucine-rich repeat protein 6 (FBXL6) has been implicated in driving cancer development across various tumor types, including hepatocellular carcinoma and colorectal cancer [16, 19]. However, its expression pattern, biological function, and mechanistic role in BLCA have yet to be elucidated.

As a key glycolytic enzyme, enolase 1 (ENO1) catalyzes the transformation of 2-phosphoglycerate to phosphoenolpyruvate in the penultimate step of glycolysis and also functions as a multifunctional regulator involved in tumor metabolism, proliferation, and migration [20, 21]. Aberrant overexpression of ENO1 has been documented in various cancers [22–26], including BLCA [27, 28], where it contributes to enhanced glycolytic activity and malignant progression [29–31]. Nevertheless, the post-translational regulation of ENO1 in BLCA remains poorly characterized, and its upstream modulators are largely unknown.

In this research, FBXL6 was found to be a notably upregulated E3 ligase in BLCA through screening multiple databases. We illustrate that FBXL6 facilitates the proliferation and migration of BLCA cells in vitro and in vivo. Overexpression of ENO1 partially restores cell proliferation, metastasis, and glycolysis that are suppressed by FBXL6 knockdown. Mechanistically, FBXL6 interacts with ENO1 and stabilizes ENO1 through K63-linked ubiquitination. The study identifies a novel FBXL6-ENO1 axis that ties ubiquitination-driven protein stabilization to metabolic reprogramming in BLCA, suggesting new therapeutic opportunities.

## Results

### FBXL6 is identified as a prognostically relevant F-box protein in BLCA

**Fig. 1:**
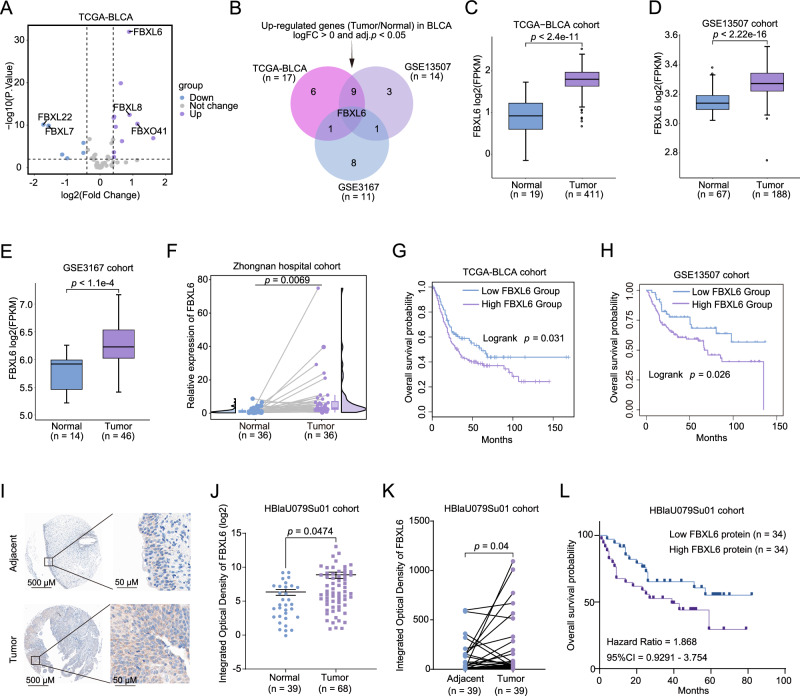
FBXL6 is upregulated in BLCA and correlates with poor prognosis. **A** Differential expression analysis of F-box genes in the TCGA-BLCA dataset. **B** Venn diagram showing FBXL6 as the only F-box gene consistently upregulated across three independent BLCA datasets (TCGA, GSE13507, and GSE3167). **C**–**E** The expression levels of *FBXL6* mRNA in BLCA tissues versus normal tissues were analyzed in TCGA (**C**), GSE13507 (**D**), and GSE3167 (**E**) cohorts. **F** *FBXL6* mRNA expression measured by qRT-PCR in 36 paired BLCA and adjacent normal tissue samples from Zhongnan Hospital of Wuhan University. **G**, **H** Kaplan–Meier survival curves showing that high FBXL6 expression is associated with poorer prognosis in TCGA (**G**) and GSE13507 (**H**) cohorts. **I** Representative IHC staining of FBXL6 in adjacent normal mucosa and tumors from the HBlaU079Su01 cohort. Scale bar = 500 μm (left); scale bar of enlarged images = 50 μm (right). **J** Quantification of FBXL6 integrated optical density in normal (*n* = 39) versus tumor (*n* = 68) specimens. **K** Paired comparison of FBXL6 IOD between matched adjacent and tumor tissues (*n* = 39). **L** Kaplan–Meier overall survival curves stratified by FBXL6 protein expression (*n* = 34 per group). Statistical significance was determined using unpaired two-tailed Student’s *t*-test (**C**–**E**, **J**), the log-rank test (**G**, **H**, **L**), and paired two-tailed Student’s *t*-test (**F**, **K**). Data are presented as mean ± SD.

To explore novel ubiquitination-related regulators in BLCA, we first assessed the prognostic value of the Cullin (CUL) protein family, which functions as a scaffold component in various types of E3 ligase complexes [32, 33] and plays pivotal roles in tumor biology [34]. Among the CUL family, only CUL1, the core scaffold of the SCF (SKP1/CUL1/F-box) complex [35–37], exhibited a significant association with poor prognosis in BLCA patients (Supplementary Fig. S1A), suggesting that the SCF complex is likely involved in the progression of BLCA. Given the critical role of F-box proteins in conferring substrate specificity within the SCF complex [38, 39], we systematically screened differentially expressed F-box family genes in BLCA across three independent datasets: TCGA, GSE13507, and GSE3167. In the TCGA cohort, FBXL6 was among the most significantly upregulated F-box genes (Fig. 1A), a finding further supported by cross-validation in the two GEO cohorts (Supplementary Fig. S1B). The intersection of these datasets identified FBXL6 as the only F-box gene consistently upregulated in BLCA (Fig. 1B), thereby highlighting it as a candidate of interest for further investigation. Subsequent analyses validated that *FBXL6* mRNA expression was notably upregulated in tumor tissues compared to normal controls across all three datasets (TCGA, GSE13507, and GSE3167) (Fig. 1C–E). To validate these findings in a real-world clinical cohort, we performed qRT-PCR on 36 paired tumor and adjacent normal tissues from BLCA patients treated at Zhongnan Hospital of Wuhan University. Compared with adjacent normal tissues, *FBXL6* mRNA expression levels were significantly elevated in tumor tissues (Fig. 1F). We next evaluated the prognostic relevance of FBXL6 in BLCA. Kaplan–Meier analysis demonstrated that high *FBXL6* mRNA expression was associated with shortened overall survival in both the TCGA and GSE13507 cohorts (Fig. 1G, H).

To further validate FBXL6 expression at the protein level, we performed IHC staining on a BLCA tissue microarray from the HBlaU079Su01 cohort. FBXL6 protein showed weak staining compared with adjacent non-cancerous tissues, and strong staining in tumors (Fig. 1I). Quantitative analysis confirmed significantly elevated FBXL6 protein levels in tumor tissues than in adjacent non-cancerous tissue (Fig. 1J). This finding was also confirmed in paired samples (Fig. 1K). Although the survival difference between high- and low-FBXL6 groups did not reach statistical significance, patients with high FBXL6 expression exhibited a clear trend toward poorer overall survival (Fig. 1L).

Furthermore, functional enrichment analysis of genes co-expressed with FBXL6 in the TCGA cohort revealed significant enrichment of pathways related to SCF-dependent proteasomal degradation and glycolytic processes (Supplementary Fig. S1C), suggesting that FBXL6 may be functionally involved in proteostasis and metabolic regulation in BLCA. Pan-cancer analysis confirms its upregulation across multiple tumor types (Supplementary Fig. S1D). While our primary focus is on its role in BLCA, this pan-cancer expression pattern suggests that novel therapeutic approaches targeting FBXL6 may find broader application.

Together, these results identify FBXL6 as a robustly upregulated and prognostically unfavorable F-box protein in BLCA, potentially involved in SCF-mediated ubiquitination and cancer metabolism.

### FBXL6 promotes BLCA cell proliferation in vitro and tumor growth in vivo

**Fig. 2:**
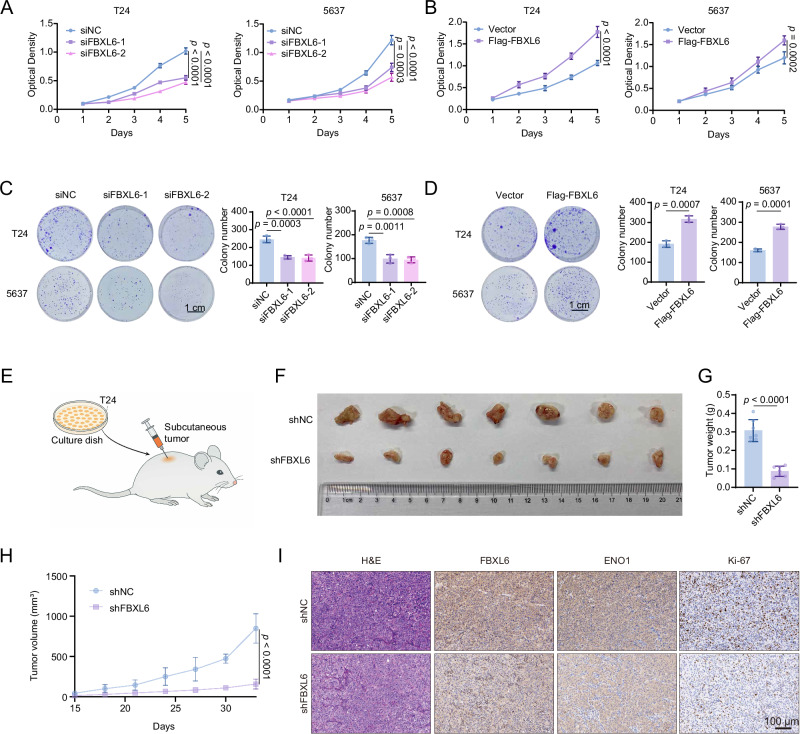
FBXL6 promotes BLCA cell proliferation in vitro and tumor growth in vivo. **A**, **B** MTT assays showing cell viability in T24 and 5637 cells transfected with siFBXL6 (**A**) or Flag-FBXL6 overexpression plasmids (**B**) over a 5-day period (*n* = 6). **C**, **D** Representative images and quantification of colony formation assays in T24 and 5637 cells following FBXL6 knockdown (**C**) or overexpression (**D**) (*n* = 3). Scale bar = 1 cm. **E** Schematic of the xenograft implantation procedure. **F**–**H** Subcutaneous xenograft model in nude mice. Representative tumor images (**F**), tumor weight (**G**), and tumor growth curve (**H**) from mice injected with shNC or shFBXL6 T24 cells (*n* = 7). **I** Immunohistochemical staining of FBXL6, ENO1, and Ki-67, along with H&E staining, in xenograft tumor sections. Scale bar = 100 μm. Data are shown as mean ± SD from three independent experiments. Statistical significance was assessed by two-tailed unpaired Student’s *t*-test (**B**, **D**, **G**, **H**) or one-way ANOVA (**A**, **C**) with Dunnett’s multiple comparison test.

To explore the functional role of FBXL6 in BLCA cell proliferation, we first manipulated FBXL6 expression in 5637 and T24 cell lines. qRT-PCR confirmed successful knockdown of *FBXL6* upon transfection with siFBXL6-1 and siFBXL6-2, while Flag-FBXL6 effectively overexpressed FBXL6 protein levels. Western blot analysis further verified corresponding changes in FBXL6 protein expression (Supplementary Fig. S2A–D). MTT assays demonstrated that siRNA-mediated knockdown of FBXL6 resulted in a marked reduction in cell viability (Fig. 2A). Conversely, FBXL6 overexpression significantly promoted cell viability over a five-day period in both 5637 and T24 cells compared to vector controls (Fig. 2B). Similarly, colony formation assays revealed that FBXL6 overexpression significantly increased the number of colonies, while FBXL6 knockdown impaired clonogenic capacity in both BLCA cell lines (Fig. 2C, D).

To confirm these results in vivo, we established a subcutaneous xenograft model using BLCA cells with stable FBXL6 knockdown or control cells. The efficiency of FBXL6 knockdown was verified through qRT-PCR and Western blot to assess mRNA and protein expression before implantation (Supplementary Fig. S2E, F). We created a diagram to visually depict the creation of the xenograft model, which involved the subcutaneous injection of BLCA cells into BALB/c Nude mice (Fig. 2E). Over time, tumor growth was observed, and tumors from the shFBXL6 group showed a significant decrease in weight and volume compared to the control group (Fig. 2F–H). Immunohistochemical analysis showed reduced Ki-67 expression, a marker of cell proliferation, in shFBXL6 tumors, corroborating in vitro results that FBXL6 promotes BLCA cell growth. In addition, the knockdown of FBXL6 resulted in lower ENO1 expression, a metabolic regulator connected to tumor proliferation (Fig. 2I).

### FBXL6 enhances BLCA cell migration in vitro and promotes metastasis in vivo

**Fig. 3:**
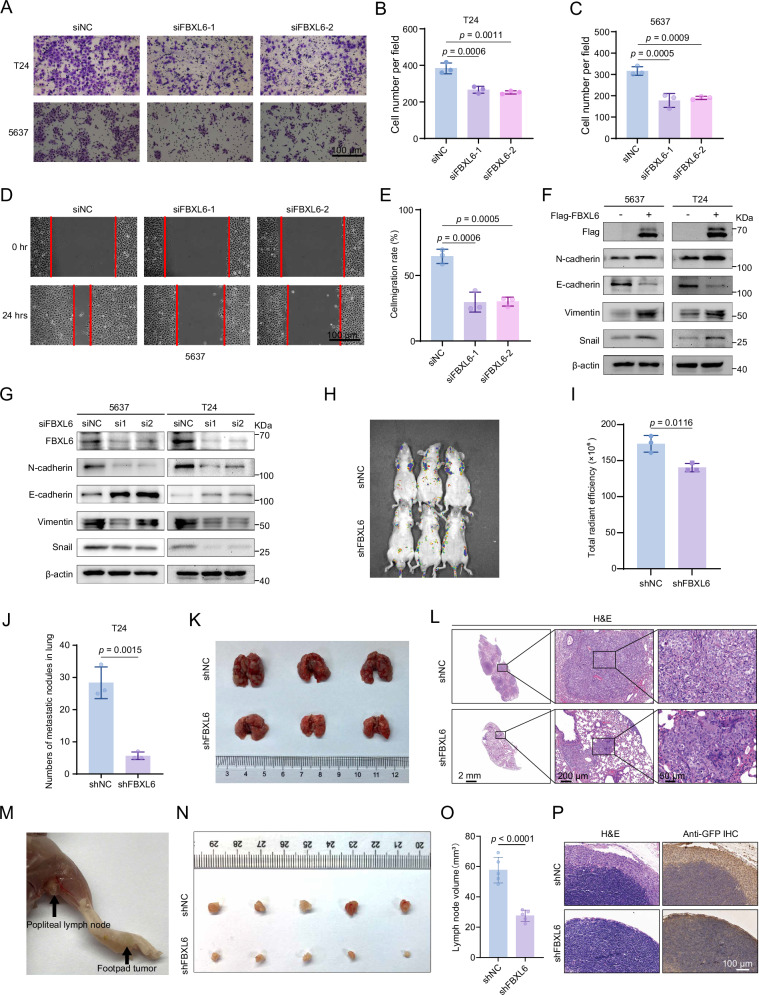
FBXL6 enhances BLCA cell migration in vitro and promotes metastasis in vivo. **A**–**C** Representative images and quantification of transwell migration assays in 5637 and T24 cells after FBXL6 knockdown using siFBXL6-1 and siFBXL6-2 (*n* = 3). Scale bar = 100 μm. **D**, **E** Wound healing assays and migration rate quantification in 5637 cells transfected with siFBXL6 or siNC (*n* = 3). Scale bar = 100 μm. **F**, **G** Western blot analysis of EMT-related markers in 5637 and T24 cells following FBXL6 overexpression (**F**) or knockdown (**G**). **H**, **I** IVIS imaging and quantification of lung metastatic burden (*n* = 3). **J**, **K** Gross lung images and quantification of metastatic nodules from lung metastasis models in T24 xenografts (*n* = 3). **L** H&E staining of lung sections showing tumor nodules. **M**–**O** Lymphatic metastasis model showing gross lymph node morphology (**M**, **N**) and volume measurements (**O**) (*n* = 5). **P** H&E staining and anti-GFP immunohistochemistry confirming tumor cell infiltration in popliteal lymph nodes. Scale bar = 100 μm. Statistical significance was assessed by two-tailed unpaired Student’s *t*-test (**I**, **J**, **O**) or one-way ANOVA (**B**, **C**, **E**) with Dunnett’s multiple comparison test.

Having observed that FBXL6 promotes the proliferation of BLCA cells, we proceeded to study its effect on cell migration. Transwell migration assays showed an increased number of migrating cells with FBXL6 overexpression (Supplementary Fig. S3A, B), whereas cells transfected with siFBXL6 had notably decreased migration compared to the siNC group (Fig. 3A–C). In wound healing experiments, overexpressing FBXL6 notably sped up wound closure after 24 h compared to the vector control (Supplementary Fig. S3C–F), while reducing FBXL6 expression significantly hindered cell movement (Fig. 3D, E and Supplementary Fig. S3G, H). We conducted a Western blot analysis of crucial proteins related to epithelial-mesenchymal transition (EMT) to investigate the mechanisms behind these observations, and demonstrated that FBXL6 overexpression led to the upregulation of mesenchymal proteins such as N-cadherin and Vimentin, along with the EMT-regulatory transcription factor Snail, while E-cadherin, an epithelial marker, showed a substantial decrease (Fig. 3F). On the other hand, reducing FBXL6 expression resulted in lower levels of Snail, N-cadherin, and Vimentin, while E-cadherin levels increased (Fig. 3G). These results imply that FBXL6 improves EMT, thus increasing the movement potential of BLCA cells. We next examined the metastatic capabilities of these cells with two in vivo models. According to IVIS imaging, gross examination, and H&E staining, mice injected with shFBXL6 cells in the lung metastasis nude mouse model had fewer metastatic nodules in their lungs than those in the control group (Fig. 3H–L). Footpad injection of shNC cells in the lymphatic metastasis model caused a considerable enlargement of the popliteal lymph nodes, but shFBXL6 knockdown markedly reduced their size (Fig. 3M–O). H&E staining of the popliteal lymph nodes revealed notable tumor cell infiltration in the shNC group, while the shFBXL6 group exhibited markedly reduced tumor presence. Immunohistochemical staining for GFP further confirmed the presence of GFP-positive tumor cells within the metastatic lesions, indicating that these nodules were derived from the implanted BLCA cells (Fig. 3P). These findings indicate that reducing FBXL6 levels inhibits the metastasis of BLCA cells in vivo, consistent with its role in promoting cell migration in vitro.

### FBXL6 positively regulates glycolysis in BLCA

**Fig. 4:**
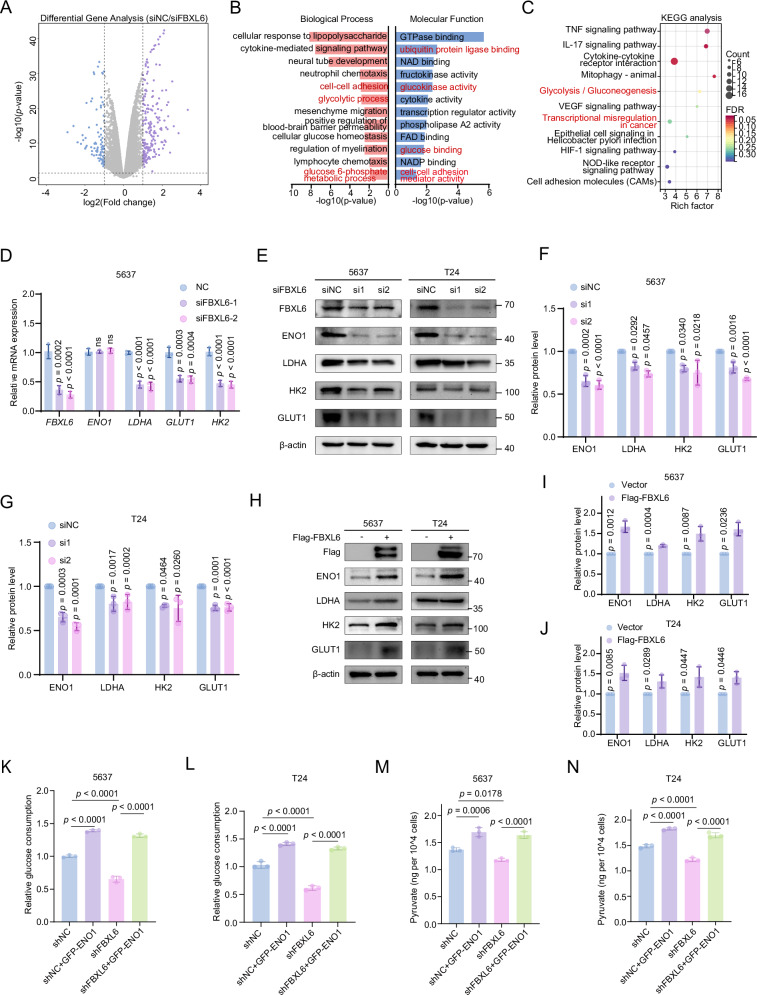
FBXL6 promotes glycolysis in BLCA cells and regulates ENO1 at the protein level. **A**–**C** RNA sequencing analysis results after FBXL6 knockdown in BLCA cells, volcano plot of differentially expressed genes (**A**), GO analysis (**B**), and KEGG analysis (**C**). **D** qRT-PCR analysis of glycolytic enzymes in *FBXL6*-knockdown 5637 (*n* = 3). **E**–**G** Western blot analysis of ENO1, LDHA, HK2, and GLUT1 protein levels following FBXL6 knockdown (**E**) and quantification of relative protein levels in 5637 (**F**) and T24 (**G**) cells (*n* = 3). **H**–**J** Western blot analysis of ENO1, LDHA, HK2, and GLUT1 protein levels following FBXL6 overexpression (**H**) and quantification of relative protein levels in 5637 (**I**) and T24 (**J**) cells (*n* = 3). **K**, **L** Glucose consumption assays in 5637 (**K**) and T24 (**L**) cells with FBXL6 knockdown and/or ENO1 overexpression (*n* = 3). **M**, **N** Pyruvate production assays in 5637 (**M**) and T24 (**N**) cells with FBXL6 knockdown and/or ENO1 overexpression (*n* = 3). Data are expressed as mean ± SD. Significance was calculated using an unpaired two-tailed Student’s *t*-test (**I**, **J**) or one-way ANOVA (**D**, **F**, **G**, **K**, **L**, **M**, **N**) followed by Dunnett’s test.

We performed RNA-seq analysis after reducing FBXL6 expression to investigate the molecular mechanisms that might allow FBXL6 to drive BLCA progression. This analysis demonstrated a significant alteration in the expression of numerous genes following FBXL6 silencing (Fig. 4A). The Gene Ontology (GO) enrichment analysis revealed a marked enrichment of pathways associated with glycolysis and ubiquitin-protein ligase activity, suggesting that FBXL6 might be involved in metabolic and post-translational regulation (Fig. 4B). This concept was further backed by KEGG pathway analysis, which identified glycolysis/gluconeogenesis as a significantly influenced pathway (Fig. 4C).

Following the knockdown of FBXL6, we assessed the expression of key glycolytic enzymes (*GLUT1*, *HK2*, *ENO1*, and *LDHA*) via qRT-PCR. These enzymes have been widely established in the field as representative markers for evaluating glycolytic activity. Previous studies commonly examine *GLUT1*, *HK2*, *ENO1*, and *LDHA* to assess glucose uptake, glycolytic commitment, intermediate conversion, and lactate production, respectively [40–42]. The mRNA levels of *LDHA*, *GLUT1*, and *HK2* showed a significant reduction, while *ENO1* mRNA remained largely not changed (Fig. 4D and Supplementary Fig. S4A). Furthermore, Western blot assays confirmed that FBXL6 knockdown reduced protein levels of LDHA, HK2, GLUT1, and ENO1, with ENO1 showing the most pronounced decrease upon FBXL6 knockdown and a corresponding increase following FBXL6 overexpression (Fig. 4E–J). Given FBXL6’s role as an E3 ubiquitin ligase regulating protein stability, and that ENO1 was the only enzyme showing discordant protein/mRNA changes among those screened, we focused subsequent experiments on ENO1.

In order to determine whether these molecular changes translated into functional metabolic consequences, we examined glucose uptake and pyruvate production in cells with FBXL6 overexpression or silencing. The knockdown of FBXL6 significantly reduced glucose consumption and pyruvate levels, whereas its overexpression increased glycolytic activity, which is consistent with the observed changes in ENO1 expression (Supplementary Fig. S4B–I). Importantly, re-expression of ENO1 in FBXL6-silenced cells largely restored glucose uptake and pyruvate production, further confirming that ENO1 is the key downstream effector molecule regulated by FBXL6 in glycolysis (Fig. 4K–N).

These findings imply that FBXL6 increases glycolytic flow in BLCA cells and could modulate important metabolic enzymes such as ENO1 through mechanisms beyond transcriptional control.

### FBXL6 interacts with ENO1 and enhances its stability via K63-linked ubiquitination

**Fig. 5:**
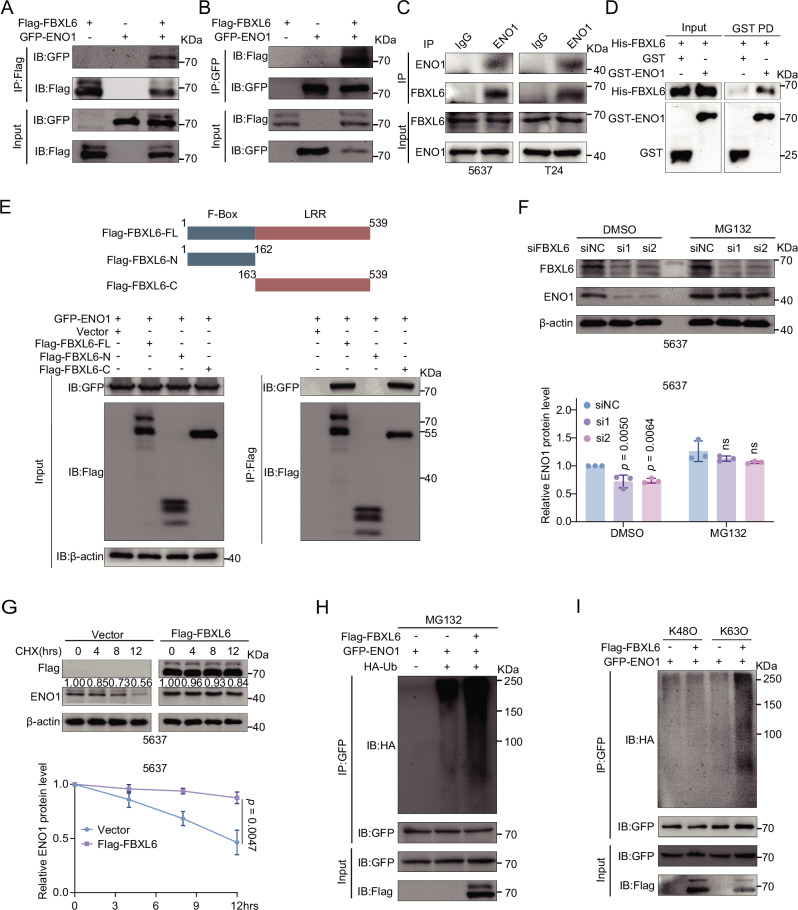
FBXL6 interacts with and stabilizes ENO1 via K63-linked ubiquitination. **A**, **B** Co-IP assays in 293T cells showing reciprocal interaction between Flag-FBXL6 and GFP-ENO1. Co-IP was performed using anti-Flag (**A**) or anti-GFP (**B**) antibodies. **C** Endogenous Co-IP analysis in 5637 and T24 cells. ENO1 antibody was used for immunoprecipitation, and FBXL6 was detected by immunoblotting. **D** GST pull-down assays were performed using purified His-FBXL6 and GST or GST-ENO1 fusion proteins. Input samples are shown as controls. **E** Schematic of FBXL6 truncation constructs (top), and Co-IP showing ENO1 interacts with the LRR-containing C-terminal domain (bottom). **F** Western blot analysis was performed to detect ENO1 protein levels in FBXL6-knockdown 5637 cells with or without MG132 treatment, and relative ENO1 levels were quantified (*n* = 3). **G** CHX chase assay assessed the effect of FBXL6 overexpression on ENO1 protein degradation and quantified the ENO1 levels. **H** Ubiquitination assays in 293T cells with Flag-FBXL6, GFP-ENO1, and HA-ubiquitin under MG132 treatment. **I** Use of K48-only and K63-only ubiquitin mutants demonstrated that FBXL6 mediates K63-linked ubiquitination of ENO1. Data are expressed as mean ± SD. Significance was calculated using an unpaired two-tailed Student’s *t*-test (**G**) or one-way ANOVA (**F**) followed by Dunnett’s test.

Since FBXL6 knockdown decreased ENO1 protein levels without notably affecting its mRNA expression, we proposed that FBXL6 might control ENO1 through post-translational processes. To investigate this possibility, we initially assessed the physical interaction between FBXL6 and ENO1. In 293T cells, Co-immunoprecipitation (Co-IP) assays showed that Flag-FBXL6 could capture GFP-ENO1 (Fig. 5A), and GFP-ENO1 could also capture Flag-FBXL6 (Fig. 5B), suggesting a mutual interaction between these proteins. We conducted immunoprecipitation with an anti-ENO1 antibody in 5637 and T24 BLCA cell lines to confirm the interaction under natural conditions. The successful Co-IP of endogenous FBXL6 confirmed the physiological significance of the interaction (Fig. 5C). To further determine whether FBXL6 directly interacts with ENO1, we performed GST pull-down assays using purified recombinant proteins (Fig. 5D). His-FBXL6 was pulled down by GST-ENO1 but not by GST alone, indicating a direct interaction between FBXL6 and ENO1.

To determine which region of FBXL6 mediates its binding to ENO1, we generated truncation constructs corresponding to the N-terminal F-box domain (1–162 aa) and the C-terminal leucine-rich repeat (LRR) domain (163–539 aa), based on the predicted structural organization of FBXL6 (Fig. 5E, top) [43]. Co-IP assays revealed that the full-length and C-terminal LRR domain of FBXL6 interacted with ENO1, whereas the F-box domain did not (Fig. 5E, bottom), suggesting that the LRR domain is responsible for substrate recognition. To further map the ENO1 region required for FBXL6 binding, we performed Co-IP experiments using ENO1 truncations. Deletion of the N-terminal region (ENO1-D2) or isolation of the C-terminal region (ENO1-D3) preserved the interaction with FBXL6, while deletion of the C-terminal portion (ENO1-D1) abolished binding (Supplementary Fig. S5A). These results indicate that the C-terminal region of ENO1 is essential for its association with FBXL6.

We next examined whether FBXL6 influences ENO1 protein stability. Treatment with the proteasome inhibitor MG132 rescued the reduction of ENO1 protein levels induced by FBXL6 depletion in both 5637 and T24 cells (Fig. 5F and Supplementary Fig. S5B, C), indicating that ENO1 undergoes proteasomal degradation. Furthermore, cycloheximide (CHX) chase assays showed that overexpression of FBXL6 significantly slowed the degradation rate of ENO1, further supporting its role in stabilizing ENO1 protein (Fig. 5G and Supplementary Fig. S5D, E).

To determine whether this regulation occurs via ubiquitination, we performed ubiquitination assays in 293 T cells co-transfected with GFP-ENO1, Flag-FBXL6, and HA-ubiquitin under MG132 treatment. Overexpression of FBXL6 markedly enhanced the ubiquitination of ENO1 (Fig. 5H). We next examined whether FBXL6 also regulates ENO1 ubiquitination at the endogenous level. Using 5637 and T24 cells treated with MG132, immunoprecipitation of endogenous ENO1 revealed a pronounced increase in ubiquitinated ENO1 upon FBXL6 overexpression (Supplementary Fig. S5F, G). Among the various polyubiquitin linkages, K48 and K63 are the most well-characterized, representing degradative and non-degradative ubiquitination pathways, respectively [44]. Therefore, we investigated the type of ubiquitin linkage involved by using K48- and K63-only HA-Ub mutants. Only the K63 mutant supported enhanced ENO1 ubiquitination in the presence of FBXL6 (Fig. 5I), indicating that FBXL6 mediates K63-linked ubiquitination, which is known to promote substrate stability rather than degradation [45].

Collectively, these findings demonstrate that FBXL6 binds to ENO1 and enhances its stability via K63-linked ubiquitination, providing a mechanistic explanation for the protein-level regulation of ENO1 observed in FBXL6-overexpressing BLCA cells.

### ENO1 is a key downstream effector of FBXL6 in BLCA

**Fig. 6:**
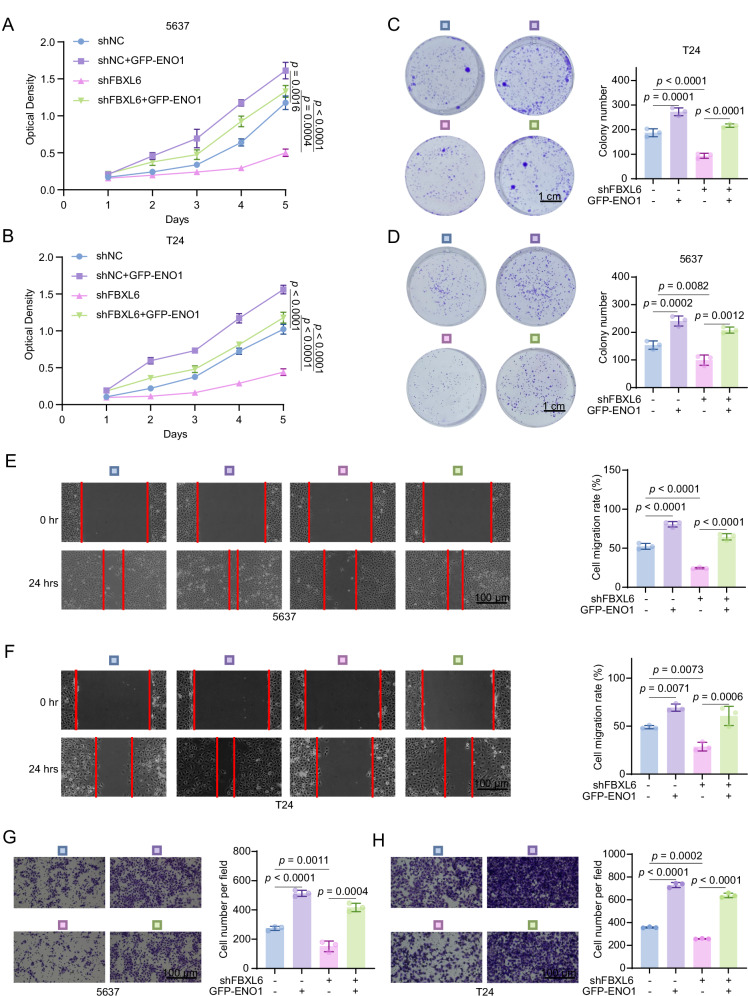
ENO1 rescues the proliferation and migration defects induced by FBXL6 knockdown in BLCA cells. **A**, **B** MTT assays in 5637 (**A**) and T24 (**B**) cells transduced with shNC, shNC + GFP-ENO1, shFBXL6, or shFBXL6 + GFP-ENO1 (*n* = 6). **C**, **D** Colony formation assays and quantification under the same four conditions in 5637 (**C**) and T24 (**D**) cells (*n* = 3). Scale bar = 1 cm. **E**, **F** Wound healing assays and quantification under the same four conditions in 5637 (**E**) and T24 (**F**) cells (*n* = 3). Scale bar = 100 μm. **G**, **H** Transwell migration assays and quantification under the same four conditions in 5637 (**G**) and T24 (**H**) cells (*n* = 3). Scale bar = 100 μm. Data are expressed as mean ± SD. Comparisons among groups were performed using one-way ANOVA (**A**–**H**) followed by Dunnett’s test.

To confirm whether ENO1 mediates the pro-tumorigenic effects of FBXL6, we conducted rescue experiments using stable knockdown of FBXL6 (shFBXL6) combined with ENO1 overexpression in BLCA cells. Four experimental groups were established: shNC, shNC + GFP-ENO1, shFBXL6, and shFBXL6 + GFP-ENO1. In MTT assays, FBXL6 knockdown significantly reduced the proliferative capacity of 5637 and T24 cells, while ectopic expression of ENO1 partially restored cell viability (Fig. 6A, B). Colony formation assays showed a similar trend, with reduced clonogenic growth upon FBXL6 knockdown and partial rescue by ENO1 (Fig. 6C, D).

Subsequently, we examined cell migration using wound healing assays in 5637 and T24 cells. The knockdown of FBXL6 greatly reduced migratory ability, but overexpressing ENO1 partially recovered the wound closure speed (Fig. 6E, F). Transwell migration assays provided additional evidence that ENO1 expression can compensate for the migration issues resulting from FBXL6 knockdown (Fig. 6G, H).

To further determine whether the oncogenic effects of FBXL6 depend on ENO1, we performed epistasis assays using ENO1 knockdown combined with FBXL6 overexpression. ENO1 depletion largely abolished the promoting effects of FBXL6 on cell proliferation, colony formation, migration, and invasion (Supplementary Fig. S6).

The results collectively imply that FBXL6 enhances BLCA cell proliferation and migration, at least partially, by controlling ENO1 protein stability, underscoring ENO1 as a significant downstream mediator of FBXL6 activity.

## Discussion

**Fig. 7:**
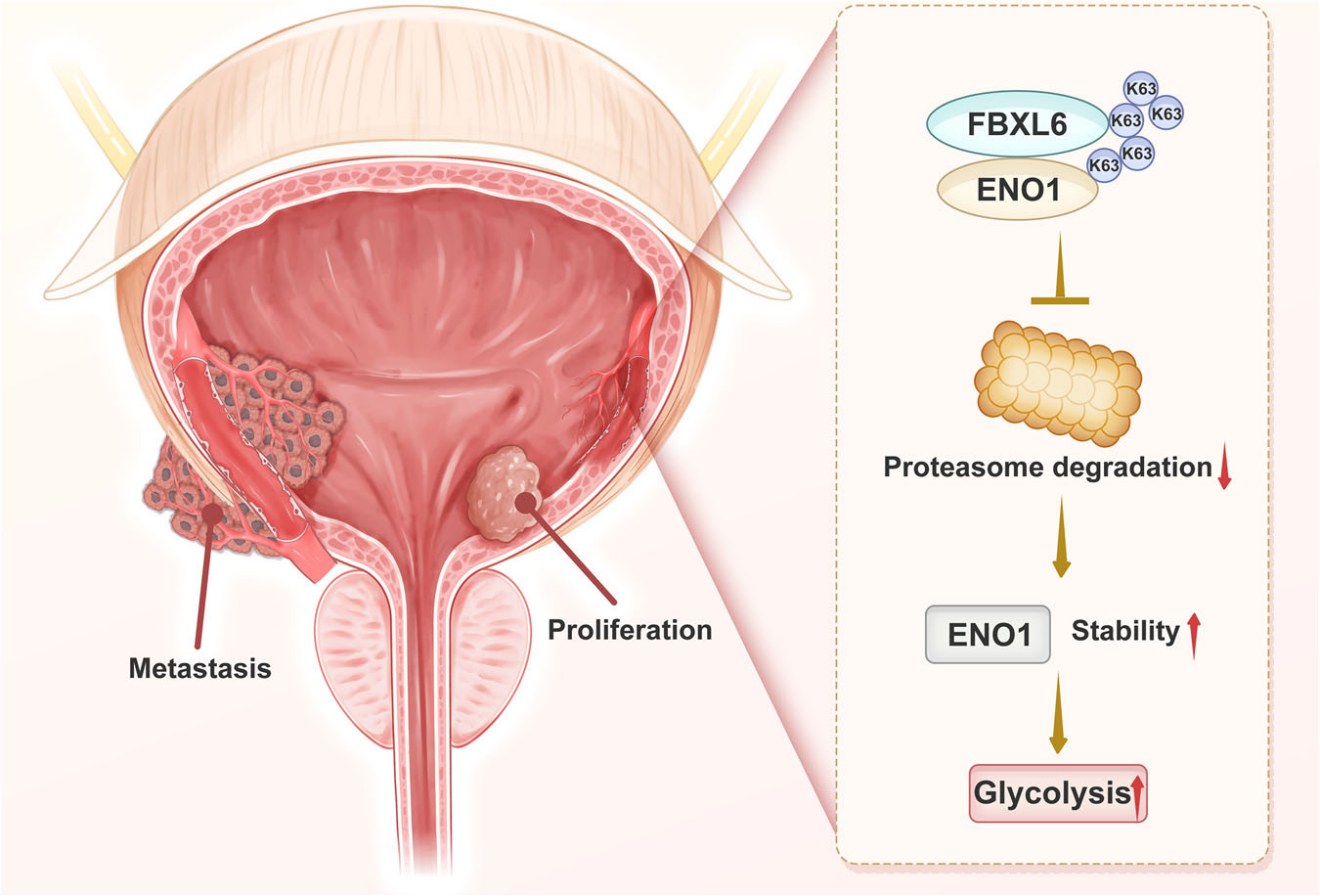
Mechanism diagram of the study. Schematic illustration of the proposed mechanism by which FBXL6 promotes BLCA progression. FBXL6 mediates K63-linked ubiquitination of ENO1, thereby enhancing its protein stability by preventing proteasomal degradation. Stabilized ENO1 facilitates glycolytic activity, contributing to increased proliferation and metastasis of BLCA cells. This graphical summary illustrates the proposed working model and is referenced in the Discussion section.

In this study, we indicated that FBXL6 is extremely upregulated in BLCA and plays a pivotal role in promoting tumor cell migration and proliferation. Overexpression of ENO1 partially restores cell proliferation, metastasis, and glycolysis that are suppressed by FBXL6 knockdown. Mechanistically, FBXL6 directly interacts with ENO1 and stabilizes ENO1 through K63-linked ubiquitination (Fig. 7). These findings not only define FBXL6 as a potential oncogenic driver in BLCA but also establish a novel link between ubiquitin-mediated post-translational regulation and metabolic reprogramming in tumor progression. These findings position FBXL6 as an upstream regulator that connects ubiquitin signaling with metabolic reprogramming, providing mechanistic insight into how cancer cells coordinate proteostasis with glycolytic control.

F-box proteins, as substrate recognition components of the SCF E3 ubiquitin ligase complex, are increasingly recognized as key modulators of cancer-related signaling pathways [43]. While FBXL6 has been implicated in several malignancies [19, 46], its role in BLCA has not been explored. Our findings expand the oncogenic spectrum of FBXL6, showing that its overexpression contributes to BLCA pathogenesis. Importantly, unlike the canonical K48-linked ubiquitination that tags proteins for proteasomal degradation, FBXL6 mediates K63-linked ubiquitination of ENO1, resulting in its stabilization rather than degradation [44, 45]. This is consistent with previous findings in colorectal cancer [47]. Together, these results support the notion that K63-linked ubiquitination may serve as a functional mechanism for modulating protein activity and stability in cancer.

ENO1 has long been considered a critical glycolytic enzyme [48–50], but its role in tumor biology extends beyond metabolism. High levels of ENO1 have been reported to correlate with poor prognosis in multiple cancers [22–24], including BLCA [27]. In our study, we showed that FBXL6-mediated ENO1 stabilization leads to increased glycolytic flux, as evidenced by increased glucose uptake, altered glycolytic metabolites, and consistent changes in glycolysis-related proteins. Although the changes in pyruvate levels observed in our study were modest in magnitude, such subtle variations are biologically expected for a terminal glycolytic metabolite whose steady-state abundance was tightly constrained by multiple downstream fates [51]. Even small shifts in pyruvate levels can indicate meaningful alterations in glycolytic flux, particularly when accompanied by consistent changes in glucose uptake, ENO1 stability, and proliferative behavior. These metabolic changes contribute directly to enhanced proliferation and migratory capacity of BLCA cells, highlighting the biological significance of the FBXL6-ENO1 axis. While ENO1 itself has been widely studied as a therapeutic target [20], our data provide evidence that its upstream regulation by FBXL6 may offer additional opportunities for intervention, particularly in targeting metabolic vulnerabilities in BLCA.

Our research also highlights the mechanistic differences in FBXL6’s role across various tumor types. For example, earlier studies showed that FBXL6 facilitates the breakdown of p53 through K-48 ubiquitination in colorectal cancer, indicating a role in degradation. Conversely, our findings indicate a non-proteolytic process in which FBXL6 stabilizes ENO1 through K63-linked chains. The differences could suggest that substrate selection is influenced by context or that cofactor interactions vary across different tumor environments. The dual role of F-box proteins might be attributed to their substrate-specific characteristics, and further research could be necessary to determine if FBXL6 controls other substrates in BLCA or associates with partners specific to certain tumor types. It is also possible that FBXL6 regulates additional substrates in BLCA, which may contribute to residual phenotypes not fully rescued by ENO1.

Despite the strength of our findings, several limitations should be acknowledged. First, although we demonstrated through truncation-based Co-IP assays that the LRR domain of FBXL6 mediates its interaction with ENO1, and that the C-terminal region of ENO1 is required for this association. However, the precise structural determinants of substrate recognition, such as individual binding residues or 3D conformational interfaces, remain to be clarified. Future studies involving mutational mapping and structural modeling may help reveal the detailed mechanism of substrate specificity. Second, while our data emphasize K63-linked ubiquitination as the major regulatory mechanism, the involvement of additional post-translational modifications or cooperating E2-conjugating enzymes was not investigated. Third, although FBXL6 and ENO1 expression levels were validated in a limited number of clinical samples, large-scale cohort studies are required to confirm their clinical significance and potential as therapeutic targets. Given that K63-linked stabilization of metabolic enzymes is emerging as a therapeutically actionable mechanism in several cancers, targeting the FBXL6-ENO1 axis may represent a complementary strategy for disrupting glycolytic dependency in BLCA.

In summary, our study reveals that FBXL6 is a novel regulator of glycolysis in BLCA, a function that is partially mediated by K63-linked ubiquitination and stabilization of ENO1. This study not only elucidates a previously uncharacterized mechanism of metabolic control in BLCA but also opens new avenues for targeted therapy by intervening in non-degradative ubiquitin signaling pathways.

## Materials and methods

### Cell lines and cell culture

Human BLCA cell lines (5637 and T24) and 293T cells were obtained from the Cell Bank of the Chinese Academy of Sciences (Shanghai, China). Cell lines were authenticated using short tandem repeat (STR) profiling and were routinely tested and confirmed to be free of mycoplasma contamination. 5637 and T24 cells were maintained in RPMI-1640 medium, and 293T cells were cultured in DMEM medium. Cells were incubated at 37 °C in a humidified atmosphere containing 5% CO₂.

### Clinical specimens

A total of 36 pairs of fresh-frozen human BLCA tissues and adjacent non-tumor tissues were collected from patients with BLCA undergoing radical cystectomy at Zhongnan Hospital of Wuhan University. All specimens were obtained with informed consent, and the study was approved by the Ethics Committee of Zhongnan Hospital (approval No. 2021125). The tissue microarray (HBlaU079Su01) (containing 68 BLCA samples and 39 adjacent non-cancerous samples) was purchased from Shanghai Outdo Biotech Co., Ltd.

### Plasmids, siRNAs, and transfection

Full-length human FBXL6 cDNA was cloned into the pcDNA3.1 vector containing an N-terminal Flag tag. Truncated constructs encoding the N-terminal (1–162 aa) and C-terminal (163–539 aa) regions of FBXL6 were subcloned into the same vector. The GFP-tagged full-length ENO1 and its truncation (D1, 1–236 aa; D2, 97–343 aa; D3, 236–343 aa) plasmids were synthesized by Miaoling Co., Ltd. (Wuhan, China). Two siRNAs targeting FBXL6 and a negative control siRNA (siNC) were synthesized by GenePharma (Shanghai, China). The sense sequences of siRNA were as follows: siFBXL6-1 (5’-GCAAGUUGUGGCUGACCUATT-3’), siFBXL6-2 (5’-GCAUCAACCGUAAUAGCAUTT-3’), and siNC (5’-UUCUCCGAACGUGUCACGUdTdT-3’).

Transient transfections were performed using Lipofectamine 3000 (Thermo Fisher Scientific) following the manufacturer’s instructions.

### RNA extraction and quantitative reverse transcription PCR (qRT-PCR)

Following the manufacturer’s instructions, total RNA was extracted using the HiPure Mini Kit (Cat. #R4111-03). cDNA amplification was performed via qRT-PCR using Bio-Rad iTaq Universal SYBR Green Supermix, and all primer sequences are detailed in Supplementary Table S1.

### Western blotting

Cell lysates were prepared using RIPA lysis buffer with protease inhibitor. Equal amounts of protein were separated by SDS-PAGE and transferred to PVDF membranes. Membranes were blocked in 5% non-fat milk and incubated with primary antibodies overnight at 4 °C. HRP-conjugated secondary antibodies were used, and signals were visualized using enhanced chemiluminescence (UVP, USA). Antibodies used in this study are detailed in Supplementary Table S2.

### Cell proliferation assays

Cell proliferation was evaluated using the MTT assay and colony formation assay. For the MTT assay, 5637 and T24 cells were plated into 96-well plates at a density of 3000 cells per well. After adherence, cells were treated accordingly and incubated for up to 5 consecutive days. At each indicated time point, 20 µL of MTT solution (Sigma) was added to each well, followed by incubation at 37 °C for 4 h. The culture medium was then carefully removed, and 200 µL of dimethyl sulfoxide (DMSO) was added to dissolve the formazan crystals. Plates were gently agitated to ensure complete solubilization, and absorbance was measured at 570 nm using a microplate reader.

For the colony formation assay, approximately 1000 cells were plated into each well of a 6-well plate and incubated for 7–10 days to allow colony formation. Afterward, colonies were fixed with 4% paraformaldehyde, stained with crystal violet (0.1%), and photographed.

### Wound healing and transwell migration assays

Wound healing assays were conducted by culturing cells in 6-well plates until ~90% confluence, followed by generating a linear scratch using a sterile 200 µL tip. Cells were gently washed with PBS to remove debris, and images were taken at 0 and 24 h.

For Transwell migration experiments, 1 × 10^5^ cells suspended in serum-free medium were loaded into the upper compartments of the inserts, while the lower chambers contained complete medium with 10% FBS as a chemoattractant. After 24 h of incubation, migrated cells adhering to the underside of the membrane were fixed, stained, and quantified under a microscope.

### Animal experiments

Stable T24 cell lines with FBXL6 knockdown (shFBXL6) or negative control (shNC) were generated via lentiviral transduction (GenePharma, Shanghai) and selected with 1 μg/mL puromycin. All animal experiments were approved by the Ethics Committee for Animal Welfare of Zhongnan Hospital of Wuhan University (Approval No. ZN2024140).

To establish subcutaneous xenografts, 1 × 10^7^ cells were suspended in 100 µL of PBS and administered subcutaneously into the right flanks of four-week-old male BALB/c nude mice. Tumor dimensions were recorded every three days with calipers, and volumes were estimated using the formula: volume = (length × width^2^)/2. On day 34, all mice were euthanized; tumors were dissected, imaged, weighed, and subjected to histopathological evaluation, including H&E and immunohistochemical staining.

For the lung metastasis model, 1 × 10^6^ cells were injected into the tail vein of nude mice (*n* = 3 per group). Six weeks post-injection, mice were subjected to in vivo imaging to monitor metastatic burden. Lungs were then harvested and subjected to gross observation, metastatic nodule counting, and H&E staining to confirm histopathological features.

For the lymph node metastasis model, 1 × 10^6^ cells in 20 μL PBS were injected into the right hind footpad. After four weeks, popliteal lymph nodes were dissected, imaged, weighed, and analyzed histologically. Immunohistochemistry using an anti-GFP antibody was performed to confirm the presence of GFP-labeled tumor cells in the metastatic lesions.

Mice were housed in a specific-pathogen-free (SPF) facility with ad libitum access to food and water. Random allocation was applied to experimental groups, and all procedures were conducted without blinding.

### Glucose uptake and pyruvate assay

Glucose consumption and pyruvate production were measured using commercial assay kits from BioVision (Glucose Uptake Assay Kit, Cat. #K606-100; Pyruvate Assay Kit, Cat. #KTB1121), following the manufacturer’s protocols.

### RNA sequencing and pathway enrichment

Following transfection of T24 cells with siNC or siFBXL6 for 48 h, total RNA was isolated using TRIzol reagent (Invitrogen) and evaluated for quality and integrity. Two microgram RNA per sample was used to construct sequencing libraries using the KCTM Stranded mRNA Library Prep Kit for Illumina^®^ (DR08402, Wuhan Seqhealth Co., Ltd., China), according to the manufacturer’s protocol. After fragment enrichment (200–500 bp), libraries were quantified and subjected to high-throughput sequencing using the DNBSEQ-T7 platform (MGI Tech Co., Ltd., China).

Raw reads were preprocessed with Trimmomatic (v0.36) to remove adapters and low-quality sequences. Cleaned reads were mapped to the human reference genome (GRCh38) using HISAT2, and gene-level expression was quantified with featureCounts (Subread v1.5.1). Differential expression analysis was carried out using the DESeq2 R package. Subsequent GO and KEGG pathway enrichment of significantly altered genes was performed using clusterProfiler (R Bioconductor). The RNA-seq data from this study have been deposited in the Gene Expression Omnibus (GEO) database with the accession number GSE295700.

### Co-IP and ubiquitination assays

Cells were lysed with IP lysis buffer and incubated with anti-Flag or anti-GFP antibodies overnight at 4 °C, followed by the BeaverBeads^TM^ Protein A/G Immunoprecipitation Kit (Cat. #22202-100, Beaver). For endogenous IP, an anti-ENO1 antibody was used. Ubiquitination assays were performed by co-transfecting Flag-FBXL6, GFP-ENO1, and HA-tagged ubiquitin into 293 T cells. MG132 (10 µM) was added 6 h before harvesting to inhibit proteasomal degradation.

### GST pull-down assay

GST pull-down assays were performed using purified recombinant proteins. GST or GST-ENO1 fusion proteins were expressed in *E*. *coli* and purified using glutathione-Sepharose beads (GE Healthcare). Bead-bound GST proteins were incubated with purified His-FBXL6 at 4 °C for 4 h with gentle rotation. After extensive washing with binding buffer (PBS supplemented with 0.1% Triton X-100), bound proteins were eluted by boiling in SDS sample buffer and subjected to SDS-PAGE followed by immunoblotting with anti-His antibody.

### Immunohistochemistry

Formalin-fixed, paraffin-embedded xenograft samples were cut into thin sections, deparaffinized, and treated for antigen retrieval. After blocking, the slides were incubated with primary antibodies at 4 °C overnight, followed by detection using horseradish peroxidase-conjugated secondary antibodies and visualization with DAB substrate. Nuclei were counterstained with hematoxylin, and the stained sections were examined under a bright-field microscope. Detailed antibody information is provided in Supplementary Table S2.

### Statistical analysis

Quantitative image analyses, including western blot band intensity, colony formation area, wound healing distance, and transwell cell migration, were conducted using ImageJ software (version 1.53). Unless specified otherwise, all quantitative data are expressed as mean ± standard deviation (SD) from at least three independent replicates. Survival analyses from TCGA and GEO datasets were performed in R software (version 4.1.3), while other statistical evaluations were carried out using GraphPad Prism (v9.0; GraphPad Software, USA). Normality was assessed using the Shapiro–Wilk test, and no dataset showed significant deviation from normal distribution. Parametric tests (Student’s *t*-test or one-way ANOVA) were therefore applied. Statistical differences between the two groups were assessed by two-tailed Student’s *t*-tests. For multiple comparisons, one-way ANOVA followed by Dunnett’s test was applied. Kaplan–Meier survival curves were compared using log-rank tests.

## Supplementary information

Supplementary Figures S1–S6

Supplementary Tables S1–S2

Original Data of Western Blots

## Data Availability

The RNA-seq data in the research have been uploaded to the GEO database with the accession code (GSE295700). TCGA-BLCA data were obtained from the UCSC Xena database (https://xena.ucsc.edu/). The GSE13507 and GSE3167 datasets were obtained from the GEO database (https://www.ncbi.nlm.nih.gov/geo/). Additional data are provided in the article or the Supplementary Information.

## References

[1] Sung H, Ferlay J, Siegel RL, Laversanne M, Soerjomataram I, Jemal A, et al. Global cancer statistics 2020: GLOBOCAN estimates of incidence and mortality worldwide for 36 cancers in 185 countries. CA Cancer J Clin. 2021;71:209–49. 33538338 10.3322/caac.21660

[2] Hanahan D, Weinberg RA. Hallmarks of cancer: the next generation. Cell. 2011;144:646–74. 21376230 10.1016/j.cell.2011.02.013

[3] Patel VG, Oh WK, Galsky MD. Treatment of muscle-invasive and advanced bladder cancer in 2020. CA Cancer J Clin. 2020;70:404–23. 32767764 10.3322/caac.21631

[4] Paul S, Ghosh S, Kumar S. Tumor glycolysis, an essential sweet tooth of tumor cells. Semin Cancer Biol. 2022;86:1216–30. 36330953 10.1016/j.semcancer.2022.09.007

[5] Ganapathy-Kanniappan S, Geschwind JF. Tumor glycolysis as a target for cancer therapy: progress and prospects. Mol Cancer. 2013;12:152. 24298908 10.1186/1476-4598-12-152 PMC4223729

[6] Meunier A, Cornet F, Campos M. Bacterial cell proliferation: from molecules to cells. FEMS Microbiol Rev. 2021;45:fuaa046. 32990752 10.1093/femsre/fuaa046 PMC7794046

[7] SenGupta S, Parent CA, Bear JE. The principles of directed cell migration. Nat Rev Mol Cell Biol. 2021;22:529–47. 33990789 10.1038/s41580-021-00366-6 PMC8663916

[8] DeBerardinis RJ, Chandel NS. Fundamentals of cancer metabolism. Sci Adv. 2016;2:e1600200. 27386546 10.1126/sciadv.1600200 PMC4928883

[9] Popovic D, Vucic D, Dikic I. Ubiquitination in disease pathogenesis and treatment. Nat Med. 2014;20:1242–53. 25375928 10.1038/nm.3739

[10] Buetow L, Huang DT. Structural insights into the catalysis and regulation of E3 ubiquitin ligases. Nat Rev Mol Cell Biol. 2016;17:626–42. 27485899 10.1038/nrm.2016.91 PMC6211636

[11] Zheng N, Shabek N. Ubiquitin ligases: structure, function, and regulation. Annu Rev Biochem. 2017;86:129–57. 28375744 10.1146/annurev-biochem-060815-014922

[12] Sampson C, Wang Q, Otkur W, Zhao H, Lu Y, Liu X, et al. The roles of E3 ubiquitin ligases in cancer progression and targeted therapy. Clin Transl Med. 2023;13:e1204. 36881608 10.1002/ctm2.1204 PMC9991012

[13] Komander D, Rape M. The ubiquitin code. Annu Rev Biochem. 2012;81:203–29. 22524316 10.1146/annurev-biochem-060310-170328

[14] Tekcham DS, Chen D, Liu Y, Ling T, Zhang Y, Chen H, et al. F-box proteins and cancer: an update from functional and regulatory mechanism to therapeutic clinical prospects. Theranostics. 2020;10:4150–67. 32226545 10.7150/thno.42735 PMC7086354

[15] Harper JW, Schulman BA. Cullin-RING ubiquitin ligase regulatory circuits: a quarter century beyond the F-box hypothesis. Annu Rev Biochem. 2021;90:403–29. 33823649 10.1146/annurev-biochem-090120-013613 PMC8217159

[16] Zhang J, Lin XT, Yu HQ, Fang L, Wu D, Luo YD, et al. Author correction: elevated FBXL6 expression in hepatocytes activates VRK2-transketolase-ROS-mTOR-mediated immune evasion and liver cancer metastasis in mice. Exp Mol Med. 2023;55:2281–6. 37783772 10.1038/s12276-023-01116-8 PMC10618234

[17] Heo J, Eki R, Abbas T. Deregulation of F-box proteins and its consequence on cancer development, progression and metastasis. Semin Cancer Biol. 2016;36:33–51. 26432751 10.1016/j.semcancer.2015.09.015 PMC4761475

[18] Lechner E, Achard P, Vansiri A, Potuschak T, Genschik P. F-box proteins everywhere. Curr Opin Plant Biol. 2006;9:631–8. 17005440 10.1016/j.pbi.2006.09.003

[19] Li Y, Cui K, Zhang Q, Li X, Lin X, Tang Y, et al. FBXL6 degrades phosphorylated p53 to promote tumor growth. Cell Death Differ. 2021;28:2112–25. 33568778 10.1038/s41418-021-00739-6 PMC8257708

[20] Capello M, Ferri-Borgogno S, Cappello P, Novelli F. α-Enolase: a promising therapeutic and diagnostic tumor target. FEBS J. 2011;278:1064–74. 21261815 10.1111/j.1742-4658.2011.08025.x

[21] Qiao G, Wu A, Chen X, Tian Y, Lin X. Enolase 1, a moonlighting protein, as a potential target for cancer treatment. Int J Biol Sci. 2021;17:3981–92. 34671213 10.7150/ijbs.63556 PMC8495383

[22] Zhang T, Sun L, Hao Y, Suo C, Shen S, Wei H, et al. ENO1 suppresses cancer cell ferroptosis by degrading the mRNA of iron regulatory protein 1. Nat Cancer. 2022;3:75–89. 35121990 10.1038/s43018-021-00299-1

[23] Li HJ, Ke FY, Lin CC, Lu MY, Kuo YH, Wang YP, et al. ENO1 promotes lung cancer metastasis via HGFR and WNT signaling-driven epithelial-to-mesenchymal transition. Cancer Res. 2021;81:4094–109. 34145039 10.1158/0008-5472.CAN-20-3543

[24] Hong J, Guo F, Lu SY, Shen C, Ma D, Zhang X, et al. *F*. *nucleatum* targets lncRNA ENO1-IT1 to promote glycolysis and oncogenesis in colorectal cancer. Gut. 2021;70:2123–37. 33318144 10.1136/gutjnl-2020-322780

[25] Xie F, Zhang H, Zhu K, Jiang CS, Zhang X, Chang H, et al. PRMT5 promotes ovarian cancer growth through enhancing Warburg effect by methylating ENO1. MedComm (2020). 2023;4:e245. 36999124 10.1002/mco2.245 PMC10044308

[26] Deng T, Shen P, Li A, Zhang Z, Yang H, Deng X, et al. CCDC65 as a new potential tumor suppressor induced by metformin inhibits activation of AKT1 via ubiquitination of ENO1 in gastric cancer. Theranostics. 2021;11:8112–28. 34335983 10.7150/thno.54961 PMC8315052

[27] Shen D, Deng Z, Liu W, Zhou F, Fang Y, Shan D, et al. Melatonin inhibits bladder tumorigenesis by suppressing PPARγ/ENO1-mediated glycolysis. Cell Death Dis. 2023;14:246. 37024456 10.1038/s41419-023-05770-8 PMC10079981

[28] Shen D, Yu X, Fan X, Liang Y, Lu D, Ke Z, et al. CDCA3-MYC positive feedback loop promotes bladder cancer progression via ENO1-mediated glycolysis. J Exp Clin Cancer Res. 2025;44:63. 39980052 10.1186/s13046-025-03325-7 PMC11841255

[29] Ma L, Xue X, Zhang X, Yu K, Xu X, Tian X, et al. The essential roles of m(6)A RNA modification to stimulate ENO1-dependent glycolysis and tumorigenesis in lung adenocarcinoma. J Exp Clin Cancer Res. 2022;41:36. 35078505 10.1186/s13046-021-02200-5 PMC8788079

[30] Xu X, Chen Y, Shao S, Wang J, Shan J, Wang Y, et al. USP21 deubiquitinates and stabilizes HSP90 and ENO1 to promote aerobic glycolysis and proliferation in cholangiocarcinoma. Int J Biol Sci. 2024;20:1492–508. 38385089 10.7150/ijbs.90774 PMC10878141

[31] Ma Q, Jiang H, Ma L, Zhao G, Xu Q, Guo D, et al. The moonlighting function of glycolytic enzyme enolase-1 promotes choline phospholipid metabolism and tumor cell proliferation. Proc Natl Acad Sci USA. 2023;120:e2209435120. 37011206 10.1073/pnas.2209435120 PMC10104498

[32] Li J, Purser N, Liwocha J, Scott DC, Byers HA, Steigenberger B, et al. Cullin-RING ligases employ geometrically optimized catalytic partners for substrate targeting. Mol Cell. 2024;84:1304–20.e1316. 38382526 10.1016/j.molcel.2024.01.022 PMC10997478

[33] Li X, Yang KB, Chen W, Mai J, Wu XQ, Sun T, et al. CUL3 (cullin 3)-mediated ubiquitination and degradation of BECN1 (beclin 1) inhibit autophagy and promote tumor progression. Autophagy. 2021;17:4323–40. 33977871 10.1080/15548627.2021.1912270 PMC8726624

[34] He ZX, Yang WG, Zengyangzong D, Gao G, Zhang Q, Liu HM, et al. Targeting cullin neddylation for cancer and fibrotic diseases. Theranostics. 2023;13:5017–56. 37771770 10.7150/thno.78876 PMC10526667

[35] Baek K, Scott DC, Henneberg LT, King MT, Mann M, Schulman BA, et al. Systemwide disassembly and assembly of SCF ubiquitin ligase complexes. Cell. 2023;186:1895–1911.e1821. 37028429 10.1016/j.cell.2023.02.035 PMC10156175

[36] Skaar JR, Pagan JK, Pagano M. SCF ubiquitin ligase-targeted therapies. Nat Rev Drug Discov. 2014;13:889–903. 25394868 10.1038/nrd4432 PMC4410837

[37] Xie CM, Wei W, Sun Y. Role of SKP1-CUL1-F-box-protein (SCF) E3 ubiquitin ligases in skin cancer. J Genet Genomics. 2013;40:97–106. 23522382 10.1016/j.jgg.2013.02.001 PMC3861240

[38] Hershko A, Ciechanover A. The ubiquitin system. Annu Rev Biochem. 1998;67:425–79. 9759494 10.1146/annurev.biochem.67.1.425

[39] Laney JD, Hochstrasser M. Substrate targeting in the ubiquitin system. Cell. 1999;97:427–30. 10338206 10.1016/s0092-8674(00)80752-7

[40] Li M, Yu J, Ju L, Wang Y, Jin W, Zhang R, et al. USP43 stabilizes c-Myc to promote glycolysis and metastasis in bladder cancer. Cell Death Dis. 2024;15:44. 38218970 10.1038/s41419-024-06446-7 PMC10787741

[41] Martínez-Ordoñez A, Seoane S, Avila L, Eiro N, Macía M, Arias E, et al. POU1F1 transcription factor induces metabolic reprogramming and breast cancer progression via LDHA regulation. Oncogene. 2021;40:2725–40. 33714987 10.1038/s41388-021-01740-6 PMC8049871

[42] Fang Y, Shen Z-Y, Zhan Y-Z, Feng X-C, Chen K-L, Li Y-S, et al. CD36 inhibits β-catenin/c-myc-mediated glycolysis through ubiquitination of GPC4 to repress colorectal tumorigenesis. Nat Commun. 2019;10:3981. 31484922 10.1038/s41467-019-11662-3 PMC6726635

[43] Skaar JR, Pagan JK, Pagano M. Mechanisms and function of substrate recruitment by F-box proteins. Nat Rev Mol Cell Biol. 2013;14:369–81. 23657496 10.1038/nrm3582 PMC3827686

[44] Tracz M, Bialek W. Beyond K48 and K63: non-canonical protein ubiquitination. Cell Mol Biol Lett. 2021;26:1. 33402098 10.1186/s11658-020-00245-6 PMC7786512

[45] Swatek KN, Komander D. Ubiquitin modifications. Cell Res. 2016;26:399–422. 27012465 10.1038/cr.2016.39 PMC4822133

[46] Zhang J, Lin XT, Yu HQ, Fang L, Wu D, Luo YD, et al. Elevated FBXL6 expression in hepatocytes activates VRK2-transketolase-ROS-mTOR-mediated immune evasion and liver cancer metastasis in mice. Exp Mol Med. 2023;55:2162–76. 37653031 10.1038/s12276-023-01060-7 PMC10618235

[47] Zhou R, Chen J, Xu Y, Ye Y, Zhong G, Chen T, et al. PRPF19 facilitates colorectal cancer liver metastasis through activation of the Src-YAP1 pathway via K63-linked ubiquitination of MYL9. Cell Death Dis. 2023;14:258. 37031206 10.1038/s41419-023-05776-2 PMC10082770

[48] Huppertz I, Perez-Perri JI, Mantas P, Sekaran T, Schwarzl T, Russo F, et al. Riboregulation of Enolase 1 activity controls glycolysis and embryonic stem cell differentiation. Mol Cell. 2022;82:2666–80.e2611. 35709751 10.1016/j.molcel.2022.05.019

[49] Zhu Q, Li J, Sun H, Fan Z, Hu J, Chai S, et al. *O*-GlcNAcylation of enolase 1 serves as a dual regulator of aerobic glycolysis and immune evasion in colorectal cancer. Proc Natl Acad Sci USA. 2024;121:e2408354121. 39446384 10.1073/pnas.2408354121 PMC11536113

[50] Hu T, Liu H, Liang Z, Wang F, Zhou C, Zheng X, et al. Tumor-intrinsic CD47 signal regulates glycolysis and promotes colorectal cancer cell growth and metastasis. Theranostics. 2020;10:4056–72. 32226539 10.7150/thno.40860 PMC7086360

[51] Prochownik EV, Wang H. The metabolic fates of pyruvate in normal and neoplastic cells. Cells. 2021;10:762. 33808495 10.3390/cells10040762 PMC8066905

