# Supplementary material for: FBXL6 promotes bladder cancer progression by stabilizing ENO1 through K63-linked ubiquitination
Source: Cell Death Discov. 2026 May 6;12:283. doi: 10.1038/s41420-026-03130-x (PMC13315011; doi:10.1038/s41420-026-03130-x)
Supplement: Supplementary file 1 — Supplementary Figures S1–S6 [file 41420_2026_3130_MOESM1_ESM.docx]

**Supplementary Figures S1-S6**


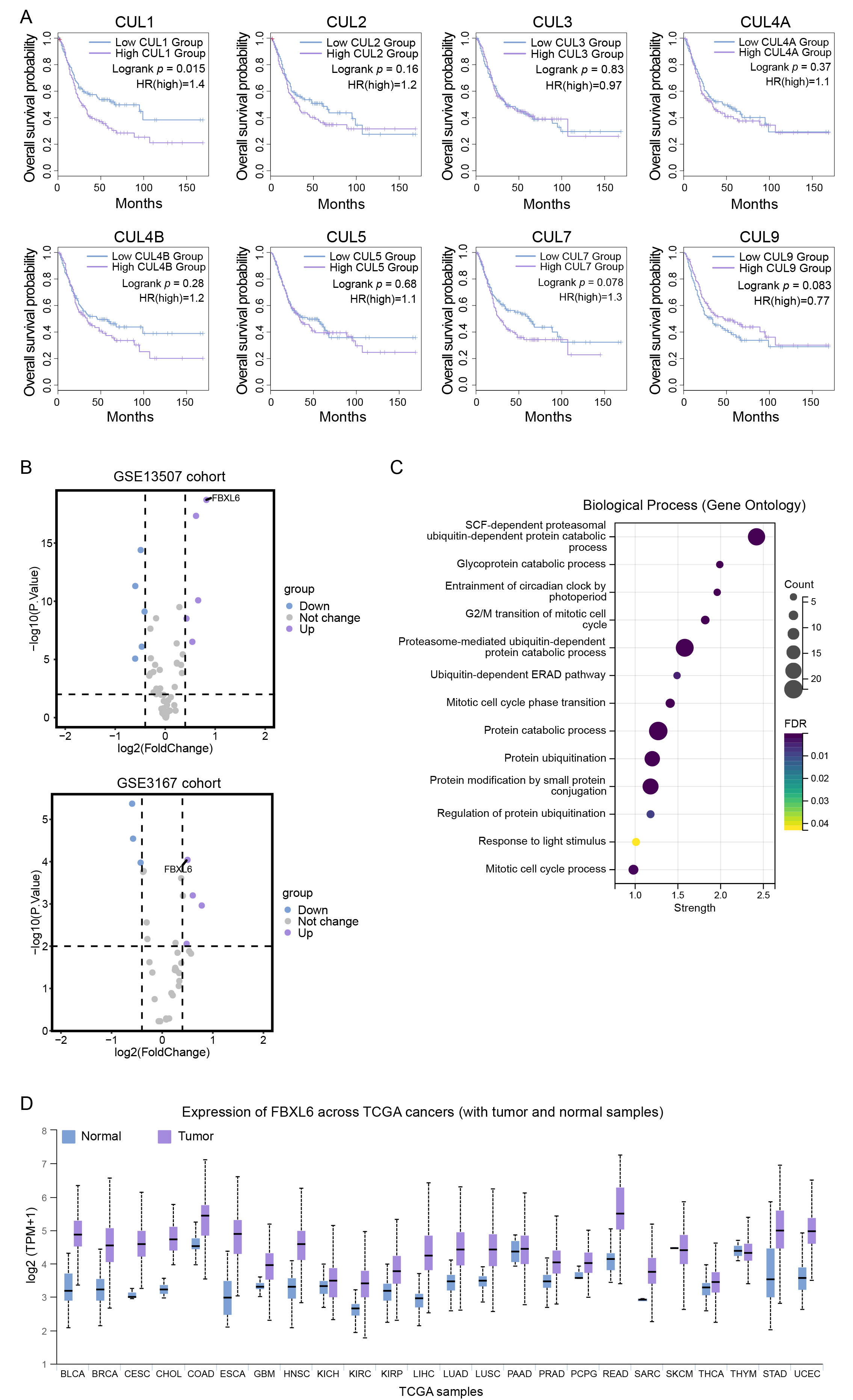


**Supplementary Figure S1. Supporting evidence for the selection of FBXL6.**

1. Overall survival analysis of CUL family genes in BLCA patients based on public datasets. **(B)** Expression heatmaps from additional databases confirming FBXL6 as consistently upregulated in BLCA. **(C)** KEGG enrichment analysis showing enrichment of SCF ubiquitination and glycolysis-related pathways. **(D)** FBXL6 expression across multiple tumor types in TCGA dataset.

**Abbreviations in the Figure S1: BLCA**: bladder urothelial carcinoma; **BRCA**: breast invasive carcinoma; **CESC**: cervical squamous cell carcinoma and endocervical adenocarcinoma; **CHOL**: cholangiocarcinoma; **COAD**: colon adenocarcinoma; **ESCA**: esophageal carcinoma; **GBM**: glioblastoma multiforme; **HNSC**: head and neck squamous cell carcinoma; **KICH**: kidney chromophobe; **KIRC**: kidney renal clear cell carcinoma; **KIRP**: kidney renal papillary cell carcinoma; **LIHC**: liver hepatocellular carcinoma; **LUAD**: lung adenocarcinoma; **LUSC**: lung squamous cell carcinoma; **PAAD**: pancreatic adenocarcinoma; **PCPG**: pheochromocytoma and paraganglioma; **PRAD**: prostate adenocarcinoma; **READ**: rectum adenocarcinoma; **SARC**: sarcoma; **SKCM**: skin cutaneous melanoma; **THCA**: thyroid carcinoma; **THYM**: thymoma; **STAD**: stomach adenocarcinoma; **UCEC**: uterine corpus endometrial carcinoma.


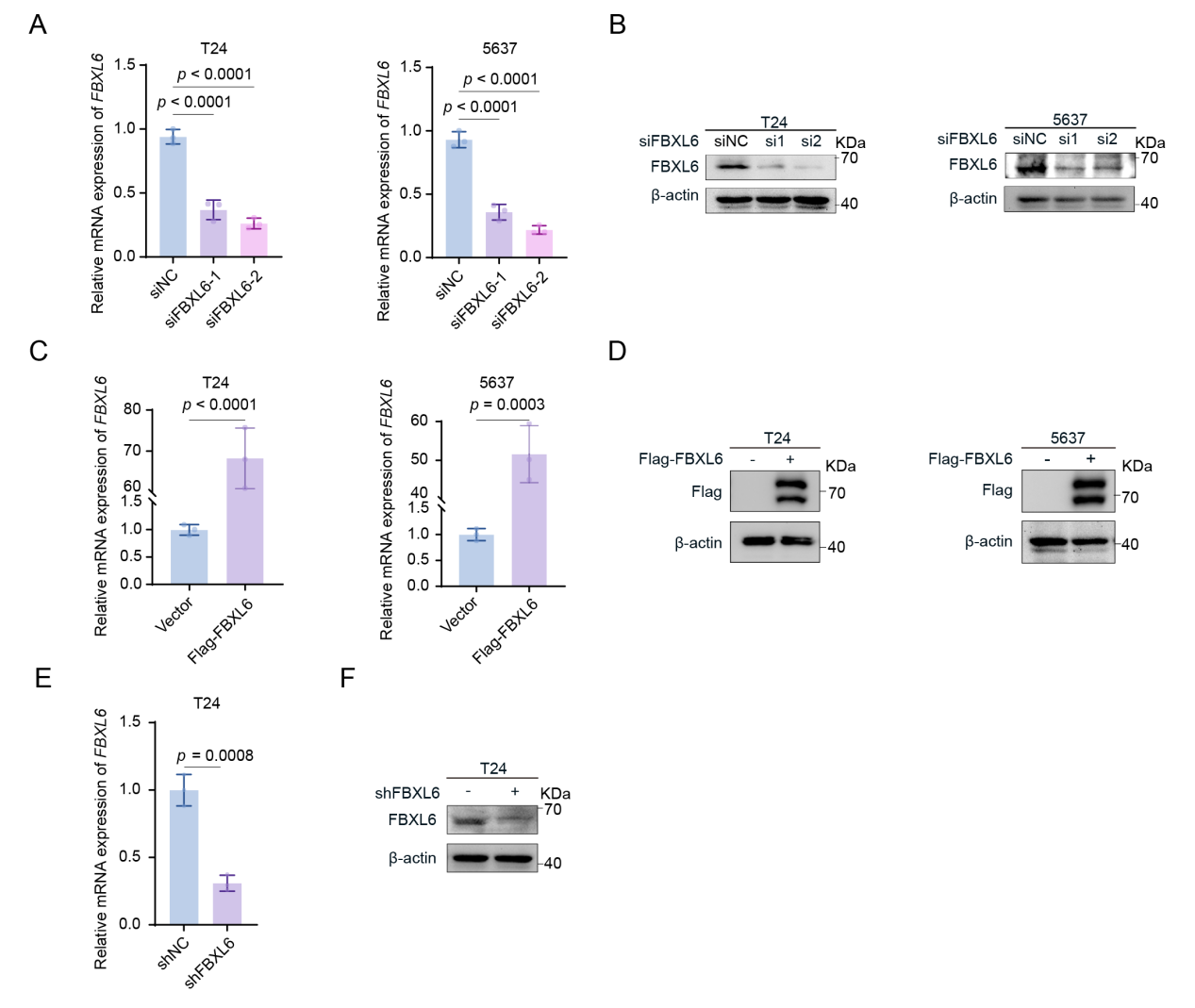


**Supplementary Figure S2. Verification of FBXL6 overexpression and knockdown efficiency in BLCA cells.**

**(A)** qRT-PCR analysis of *FBXL6* mRNA levels in T24 and 5637 cells transfected with siFBXL6-1, siFBXL6-2, or siNC (n = 3). **(B)** Western blot analysis of FBXL6 protein levels in T24 and 5637 cells following siRNA-mediated knockdown**. (C)** qRT-PCR analysis of *FBXL6* mRNA levels in T24 and 5637 cells transfected with Flag-FBXL6 or vector plasmids (n = 3). **(D)** Western blot analysis of Flag-FBXL6 expression in T24 and 5637 cells. **(E-F)** qRT-PCR and Western blot validation of FBXL6 knockdown efficiency in stably transduced T24 cells prior to tumor implantation (n = 3). The data are shown as the means ± SDs. *p*-values were determined by two-tailed unpaired Student’s t-test (C and E) and one-way ANOVA with Dunnett’s multiple comparisons (A).


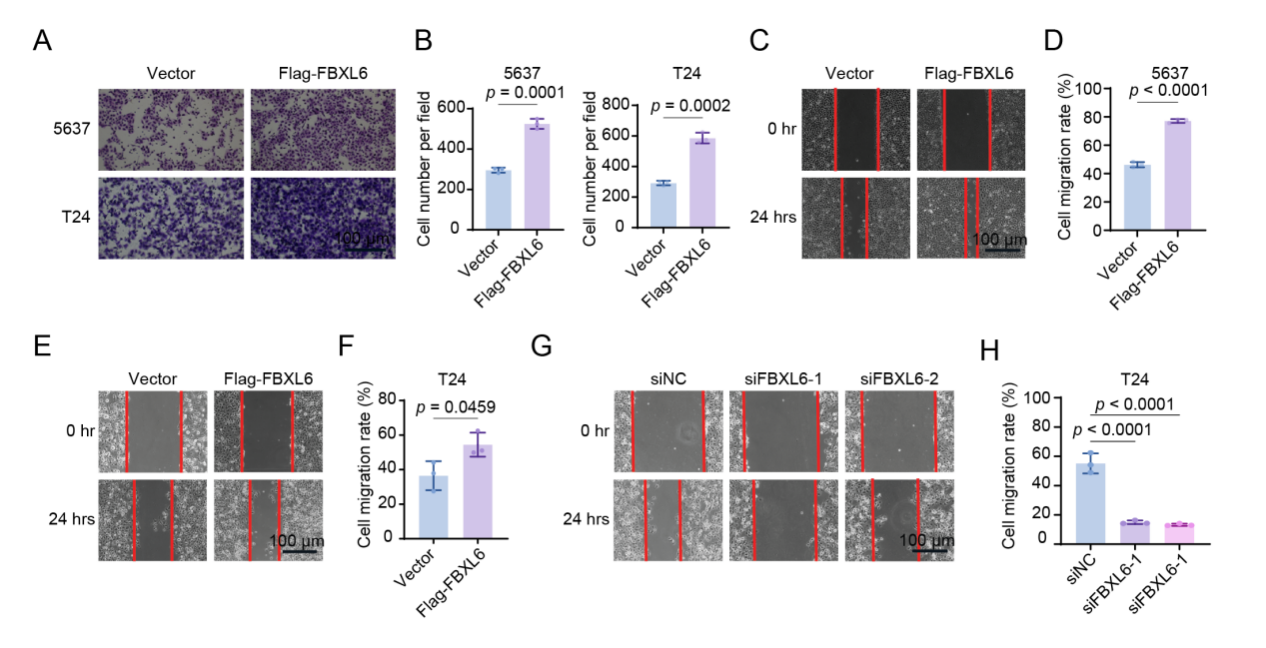


**Supplementary Figure S3. FBXL6 promotes BLCA cell migration *in vitro*.**

**(A-B)** Transwell migration assays (A) and quantification (B) in 5637 and T24 cells transfected with Flag-FBXL6 or vector plasmids (n = 3). Scale bar = 100 μm. **(C-F)** Wound healing assays and quantification in 5637 (C-D) and T24 (E-F) cells overexpressing FBXL6 or vector control (n = 3). Scale bar = 100 μm. **(G-H)** Wound healing assays (G) and quantification (H) in T24 cells transfected with siFBXL6 or siNC (n = 3). Scale bar = 100 μm. The data are shown as the means ± SDs. The *p*-values were determined by two-tailed unpaired Student’s t-test (B, D, and F) and one-way ANOVA followed by Dunnett’s multiple comparisons (H).


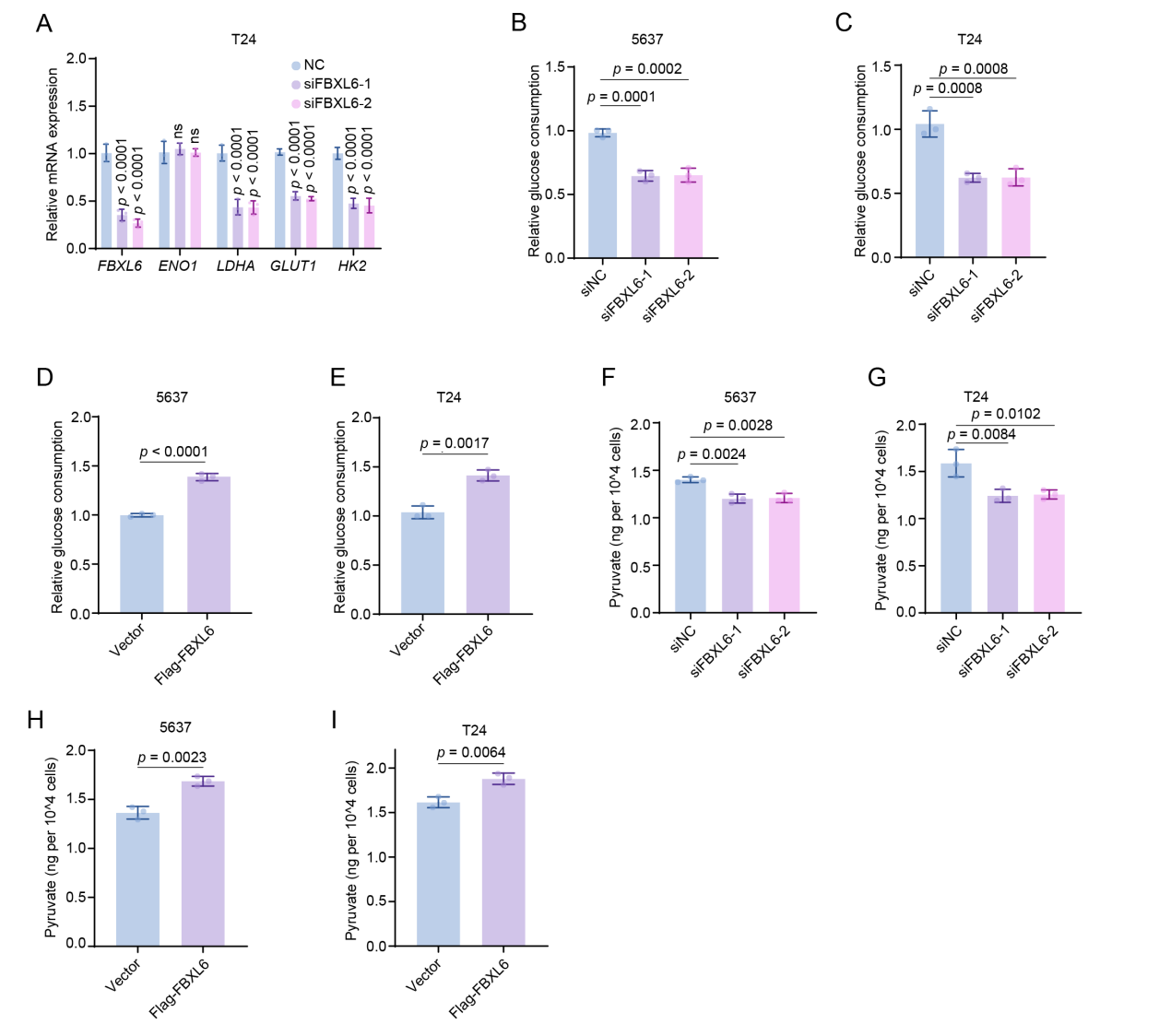


**Supplementary Figure S4. FBXL6 promotes glycolysis in BLCA cells.**

**(A)** qRT-PCR analysis of glycolytic enzymes in FBXL6 knockdown T24 cells (n = 3). **(B-C)** Glucose consumption assays were performed in 5637 (B) and T24 (C) cells transfected with siFBXL6 (n = 3). **(D-E)** Glucose consumption assays were performed in 5637 (D) and T24 (E) cells transfected with Flag-FBXL6 and vector plasmids (n = 3). **(F-G)** Pyruvate production assays were performed in 5637 (F) and T24 (G) cells transfected with siFBXL6 (n = 3). **(H-I)** Pyruvate production assays were performed in 5637 (H) and T24 (I) cells transfected with Flag-FBXL6 and vector plasmids (n = 3). The data are shown as the means ± SDs. The *p*-values were determined by one-way ANOVA followed by Dunnett’s multiple comparisons (A, B, C, F, and G) and two-tailed unpaired Student’s t-test(D, E, H, and I).


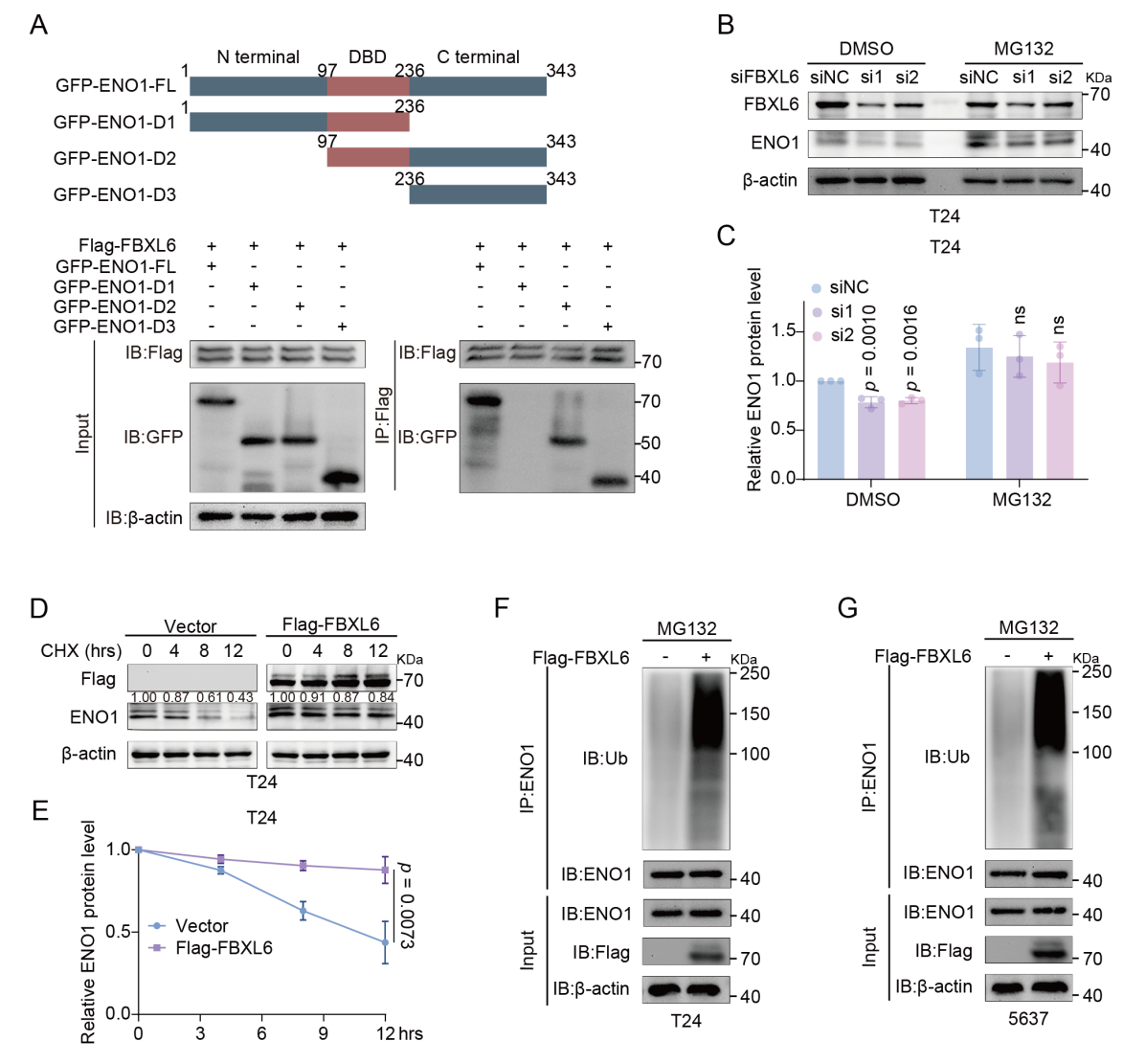


**Supplementary Figure 5. FBXL6 interacts with and stabilizes ENO1 via K63-linked ubiquitination.**

**(****A)** Schematic of ENO1 truncation constructs (top), and Co-IP showing FBXL6 interacts with C-terminal domain of ENO1 (bottom). **(B-C)** Western blot analysis was performed to detect ENO1 protein levels in FBXL6-knockdown T24 cells with or without MG132 treatment (B), and relative ENO1 levels were quantified (n = 3) (C). **(D-E)** CHX chase assay assessed the effect of FBXL6 overexpression on ENO1 protein degradation and quantified the ENO1 levels. **(F-G)** Ubiquitination assays were performed in T24 (F) and 5637 (G) cells transfected with Flag-FBXL6 under MG132 treatment. The data are shown as the means ± SDs. The *p*-values were determined by two-tailed unpaired Student’s t-test (E) and one-way ANOVA followed by Dunnett’s multiple comparisons (C).


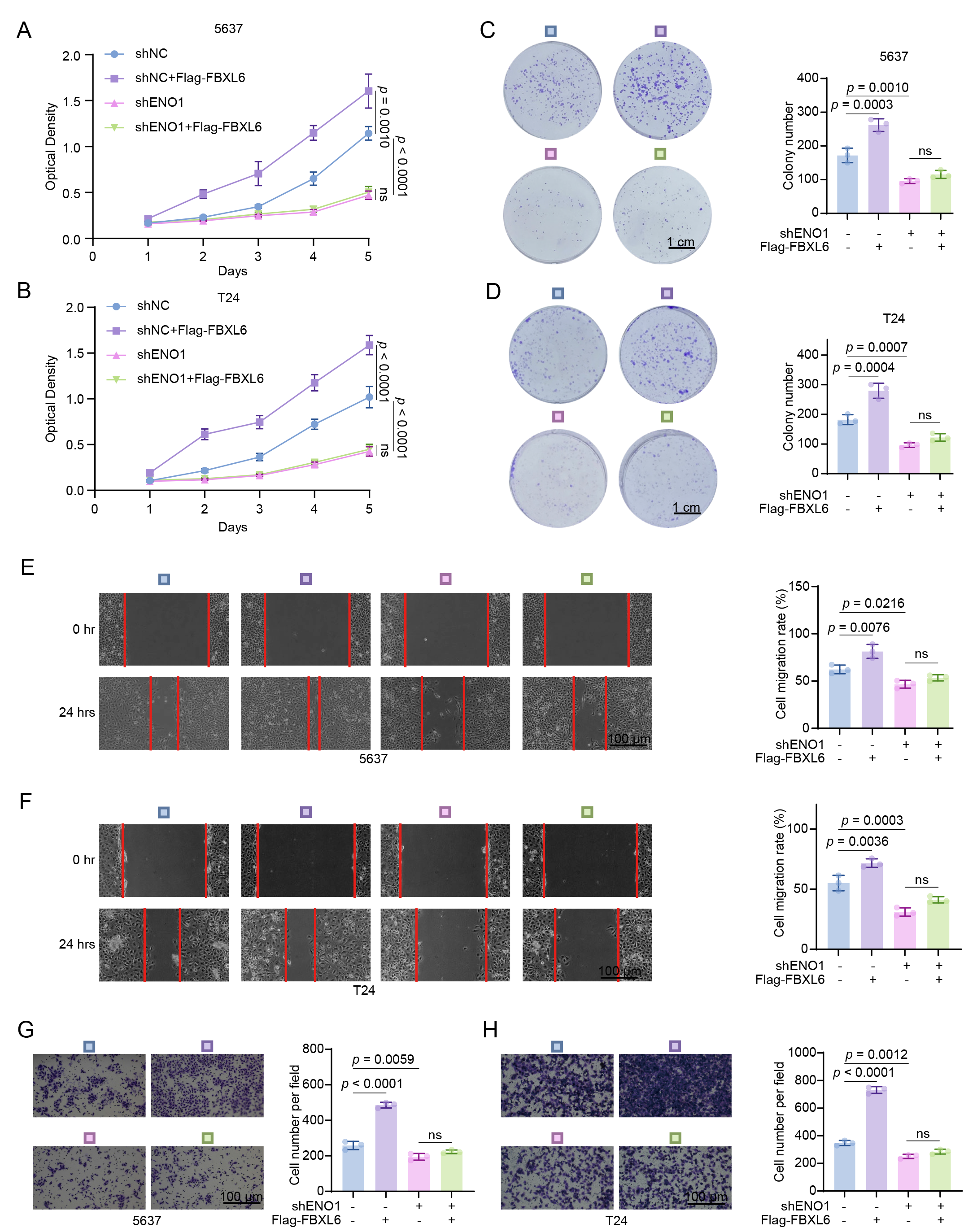


**Supplementary Figure 6. FBXL6-mediated malignant phenotypes are largely dependent on ENO1 in bladder cancer cells.**

**(A-B)** MTT assays in 5637 (A) and T24 (B) cells transduced with shNC, shNC + Flag-FBXL6, shENO1, or shENO1 + Flag-FBXL6 (n = 6). **(C-D)** Colony formation assays and quantification under the same four conditions in 5637 (C) and T24 (D) cells (n = 3). Scale bar = 1 cm. **(E-F)** Wound healing assays and quantification under the same four conditions in 5637 (E) and T24 (F) cells (n = 3). Scale bar = 100 μm. **(G-H)** Transwell migration assays and quantification under the same four conditions in 5637 (G) and T24 (H) cells (n = 3). Scale bar = 100 μm. Data are expressed as mean ± SD. Comparisons among groups were performed using one-way ANOVA (A, B, C, D, E, F, G, and H) followed by Dunnett’s test.
