# Supplementary material for: FBXL6 promotes bladder cancer progression by stabilizing ENO1 through K63-linked ubiquitination
Source: Cell Death Discov. 2026 May 6;12:283. doi: 10.1038/s41420-026-03130-x (PMC13315011; doi:10.1038/s41420-026-03130-x)
Supplement: Supplementary file 2 — Supplementary Tables S1–S2 [file 41420_2026_3130_MOESM2_ESM.docx]

**Supplementary Tables S1-S2**

**Supplementary Table S1.** Primers used in qRT-PCR assays.

| **Gene** | **Forward Primer (5’>3’)** | **Reverse Primer (5’>3’)** |
| --- | --- | --- |
| *FBXL6* | GGATCTTCGTGGCTGTGC | CATACAGGCCCAGATGAAGC |
| *β-actin* | GATCCACATCTGCTGGAAG | CAGCACAATGAAGATCAAGA |
| *ENO1* | GTTCACAGCCAGTGCAGGAA | GGAGGCAGTTGCAGGACTTC |
| *GLUT1* | CTTTGTGGCCTTCTTTGAAGT | CCACACAGTTGCTCCACAT |
| *HK2* | GAGCCACCACTCACCCTACT | CCAGGCATTCGGCAATGTG |
| *LDHA* | ACGTGCATTCCCGATTCCTT | GGAAAAGGCTGCCATGTTGG |

**Supplementary Table S2.** Details of antibodies.

| **Protein** | **Catalog No.** | **Source** | **Dilution or amount** |
| --- | --- | --- | --- |
| GFP | SC-9996 | Santa Cruz | IP/1 μg, WB/1:1000, IHC/1:200 |
| GFP | AE012 | Abclonal | WB/1:2000 |
| Flag | F1804 | Sigma | IP/1 μg, WB/1:1000 |
| Flag | 20543-1-AP | Proteintech | IP/1 μg, WB/1:1000 |
| HA | TA180128 | Origene | IP/1 μg, WB/1:1000 |
| GST | 10000-0-AP | Proteintech | WB/1:1000 |
| His | 66005-1-lg | Proteintech | WB/1:1000 |
| ENO1 | ab227978 | Abcam | IP/1 μg, WB/1:1500, IHC/1:200 |
| FBXL6 | bs-16041R | Bioss | WB/1:1000, IHC/1:200 |
| lgG | B900610 | Proteintech | IP/1 μg, WB/1:1000 |
| β-actin | 66009-1-Ig | Proteintech | WB/1:1000 |
| Vimentin | 5741S | Cell Signaling | WB/1:1000 |
| snail | 3879S | Cell Signaling | WB/1:1000 |
| E-Cadherin | 3195S | Cell Signaling | WB/1:5000 |
| Ki67 | ab16667 | Abcam | IHC/1:200 |
| N-cadherin | 22018-1-AP | Proteintech. | WB/1:2000 |
| LDHA | 3582 | Cell Signaling | WB/1:1000 |
| HK2 | 22029-1-AP | Proteintech | WB/1:1000 |
| Ubiquitin | sc-8017 | Santa Cruz | WB/1:1000 |
| GLUT1 | 21829-1-AP | Proteintech | WB/1:1000 |

IP: Immunoprecipitation

WB: Western blot

IHC: Immunohistochemistry
