# Supplementary material for: FBXL6 promotes bladder cancer progression by stabilizing ENO1 through K63-linked ubiquitination
Source: Cell Death Discov. 2026 May 6;12:283. doi: 10.1038/s41420-026-03130-x (PMC13315011; doi:10.1038/s41420-026-03130-x)

Figure 3F

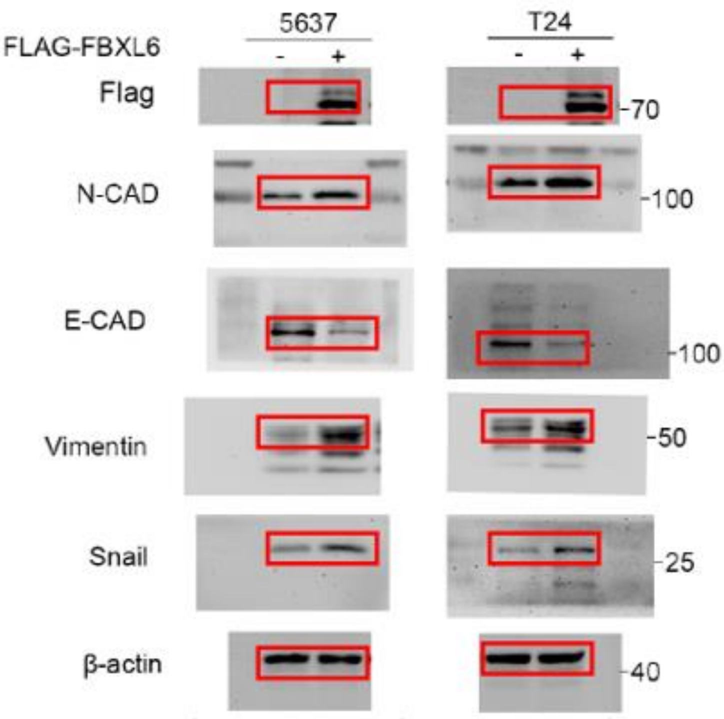

Figure 3G

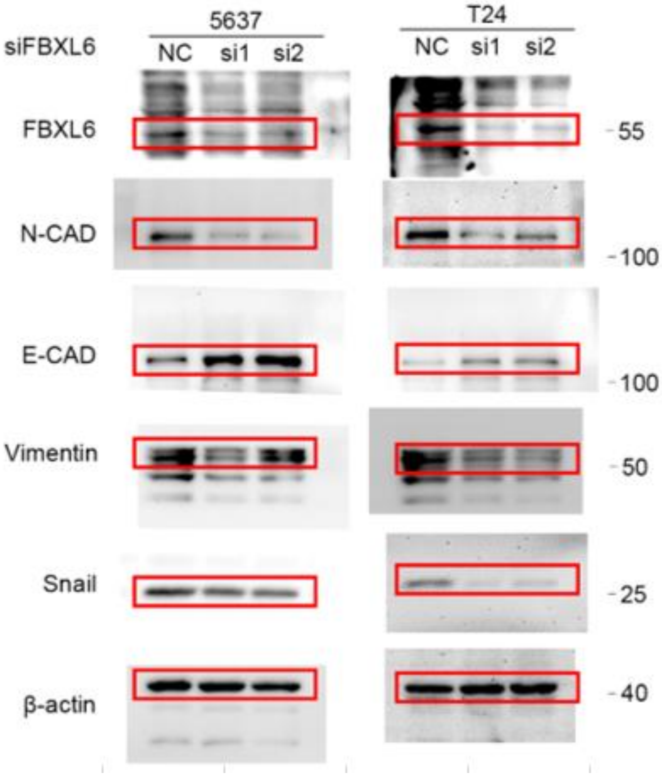

Figure 4E

Experiment 1

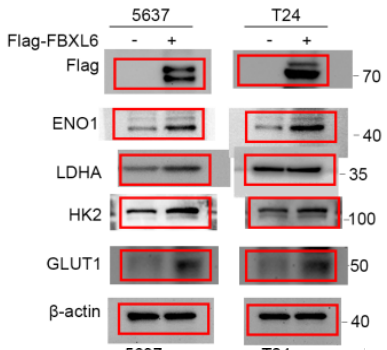

Experiment 2

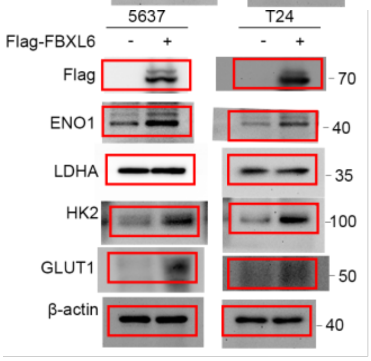

Experiment 3

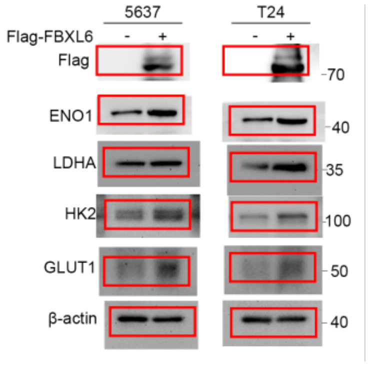

Figure 4H

Experiment 1

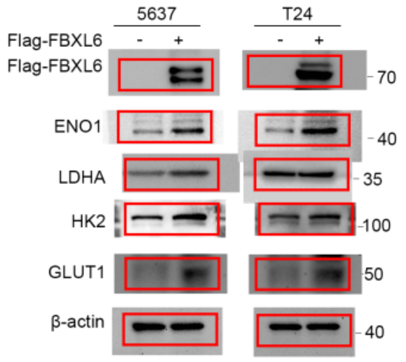

Experiment 2

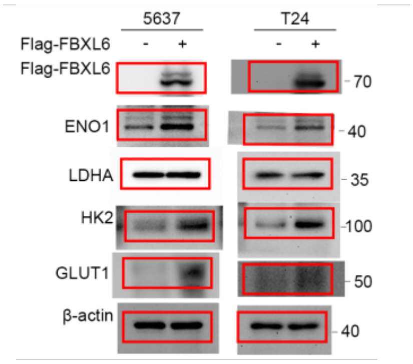

Experiment 3

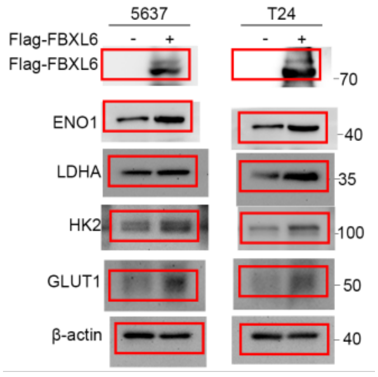

Figure 5A

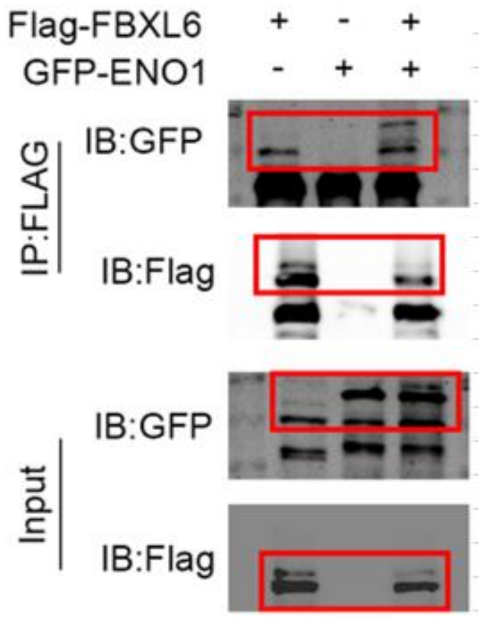

Figure 5B

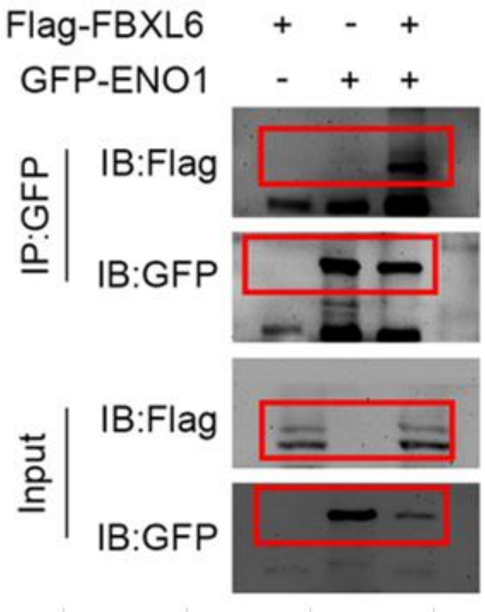

Figure 5C

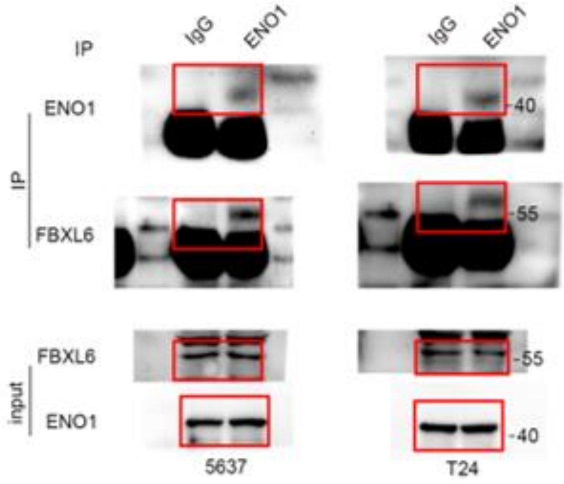

Figure 5D

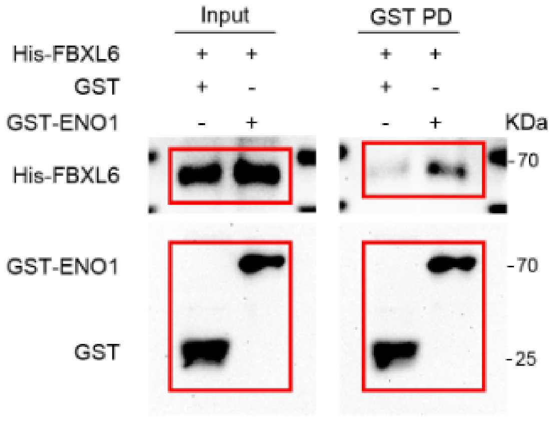

Figure 5E

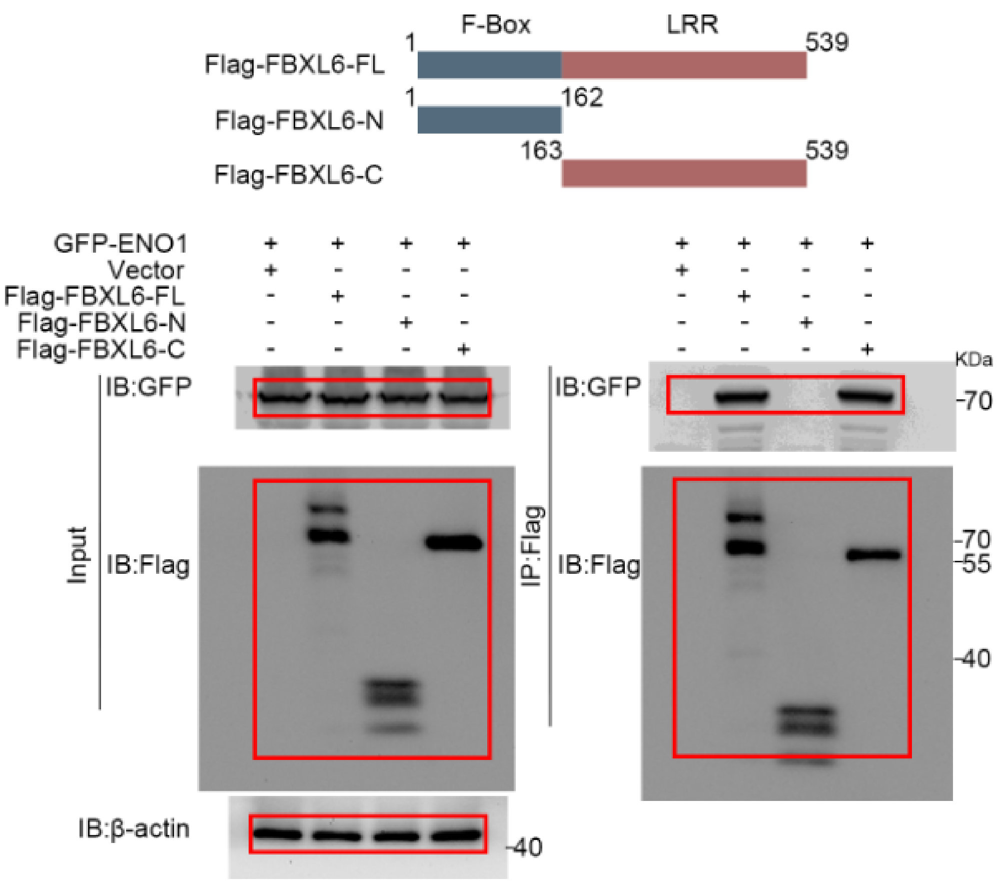

Figure 5F

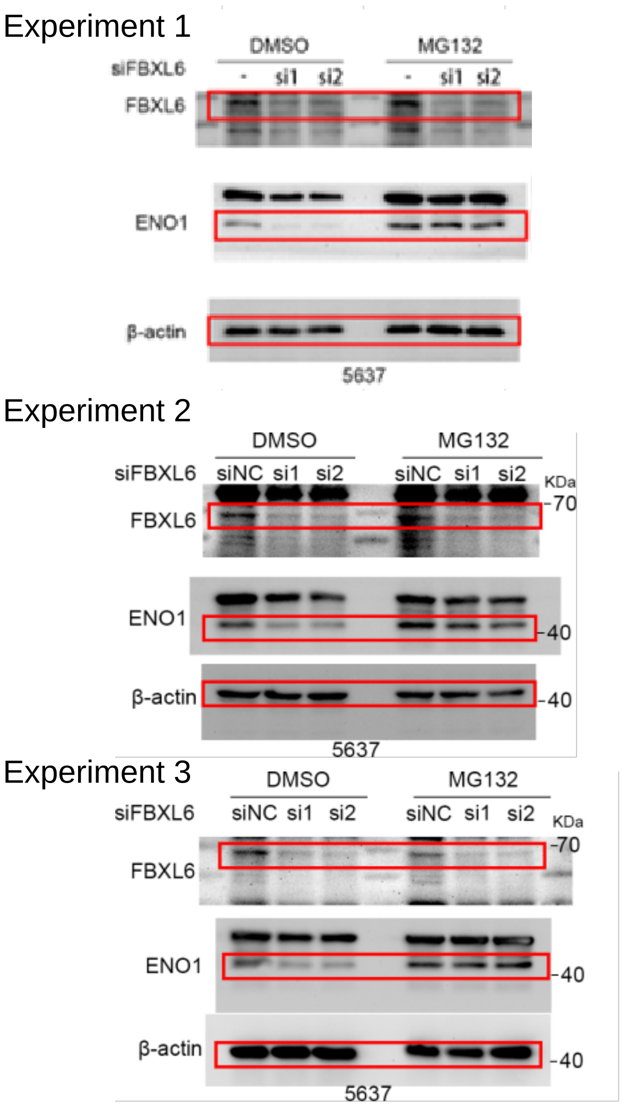

Figure 5G

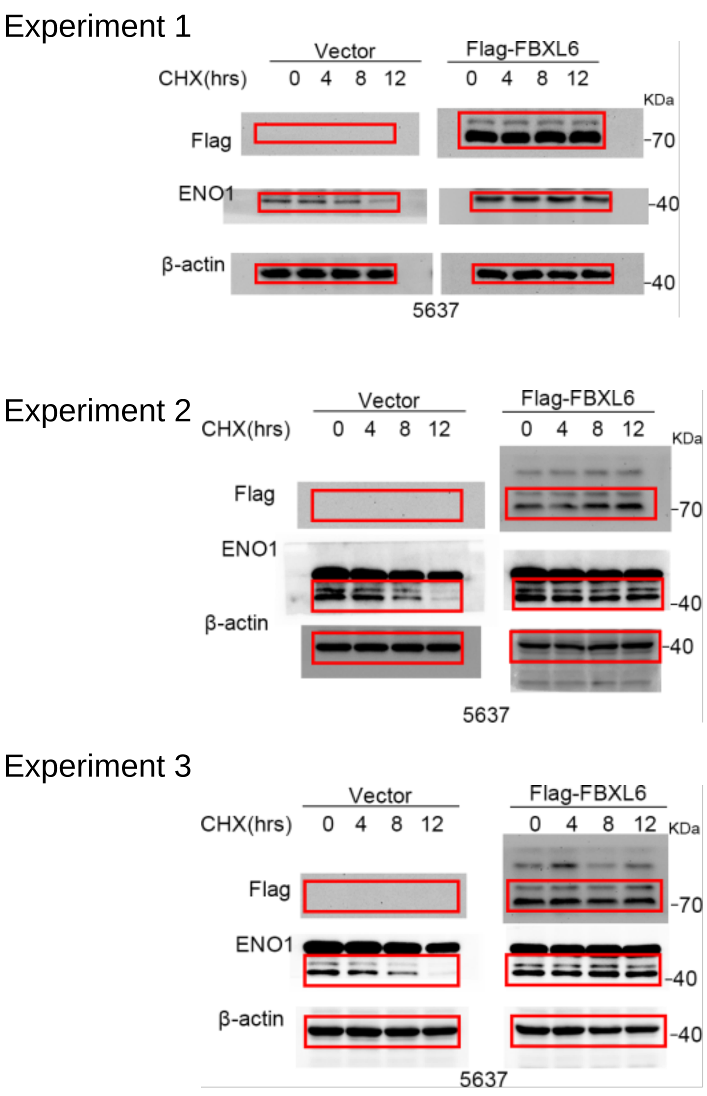

Figure 5H

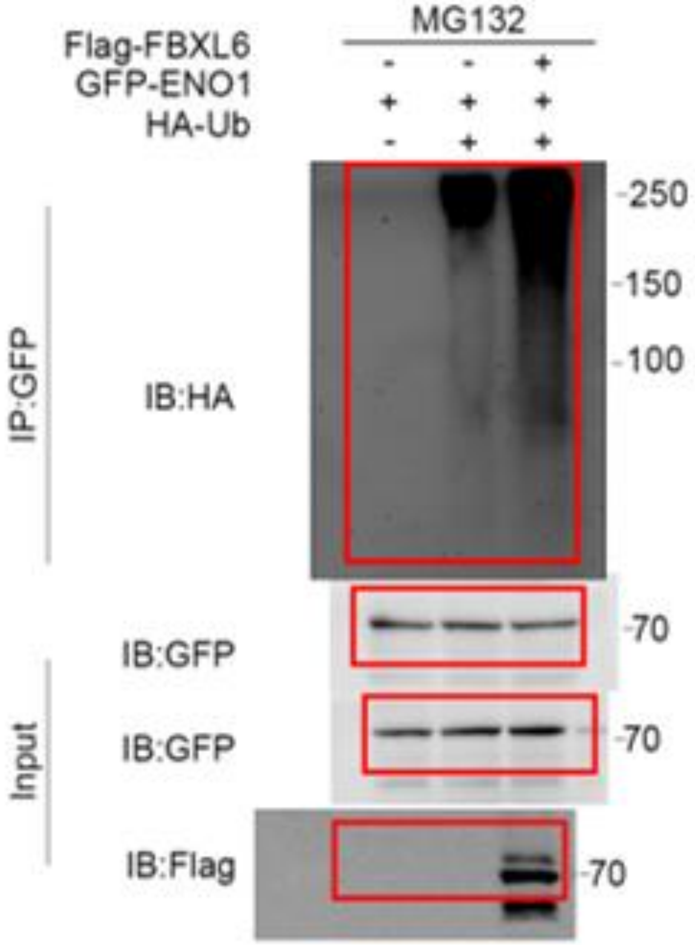

Figure 5I

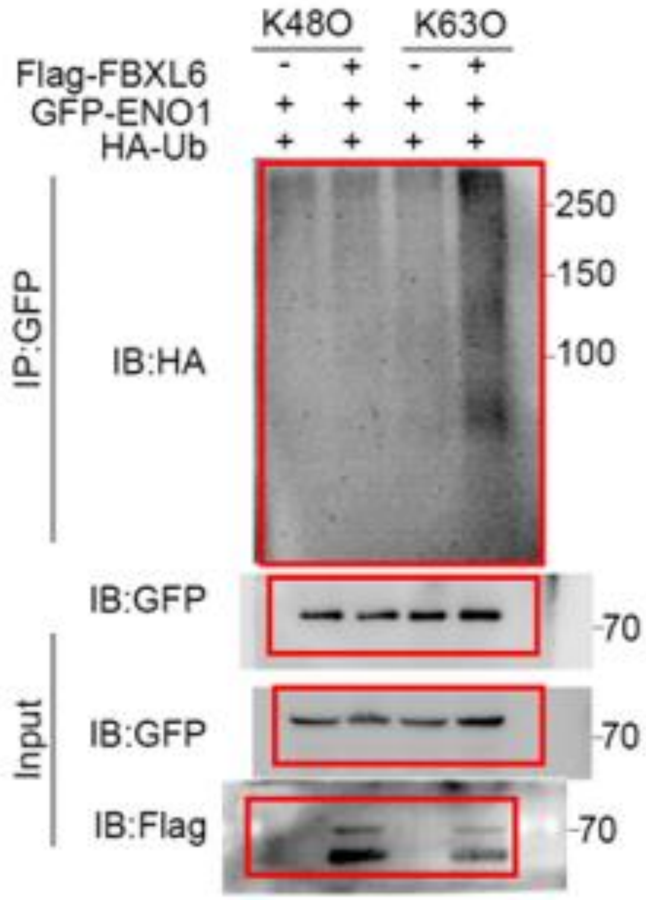

Supplementary Figure 2B

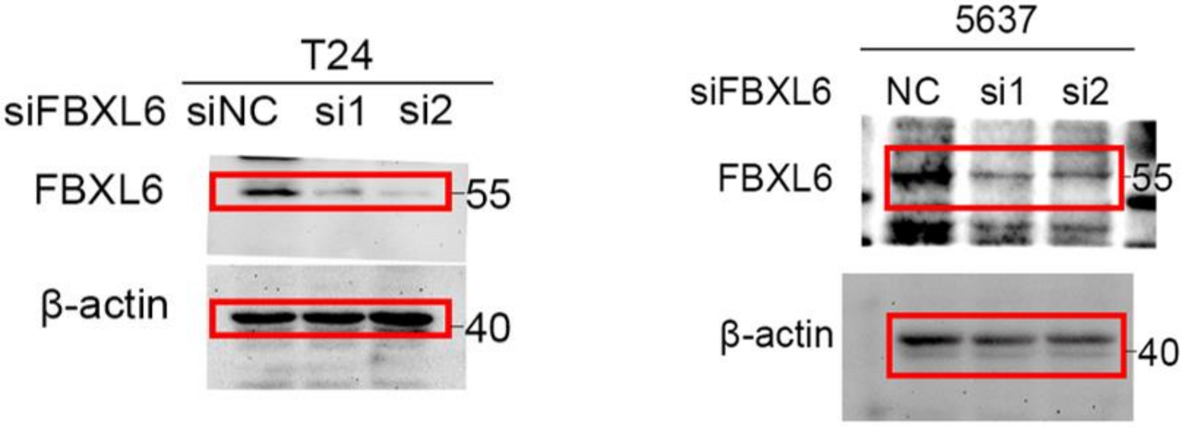

Supplementary Figure 2D

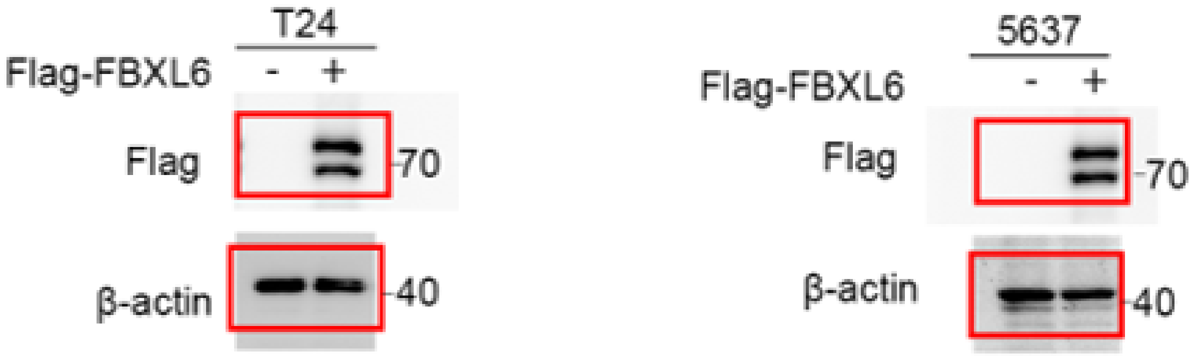

Supplementary Figure 2F

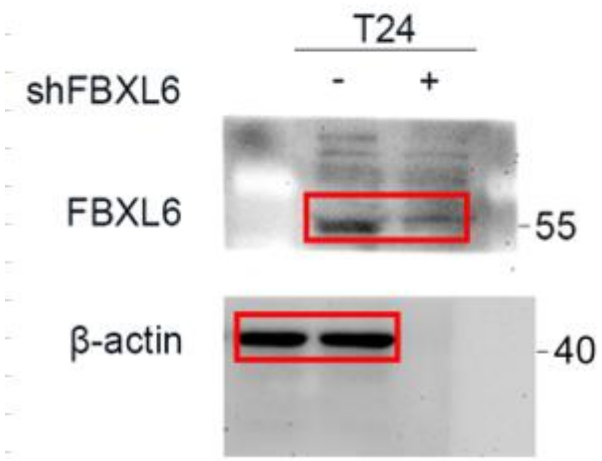

Supplementary Figure 5A

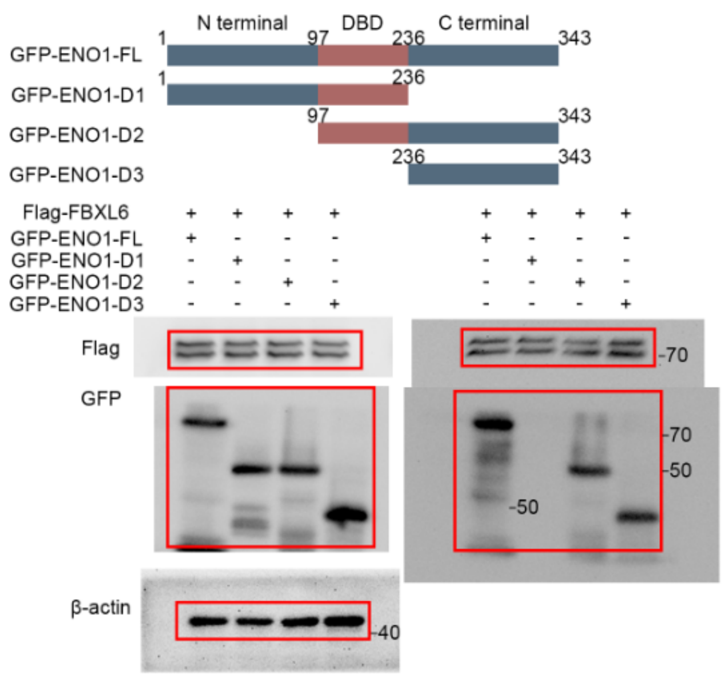

Supplementary Figure 5B

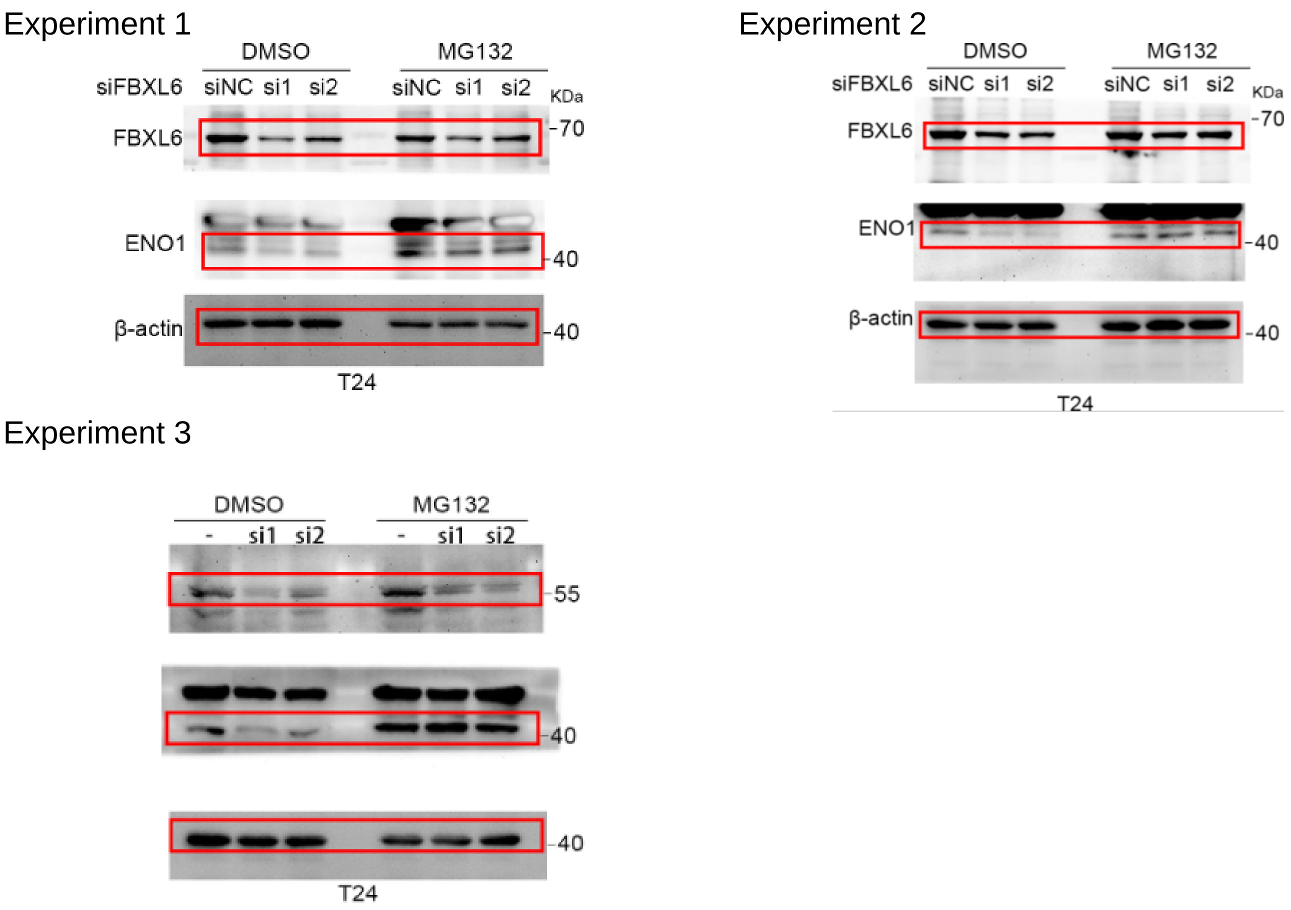

Supplementary Figure 5D

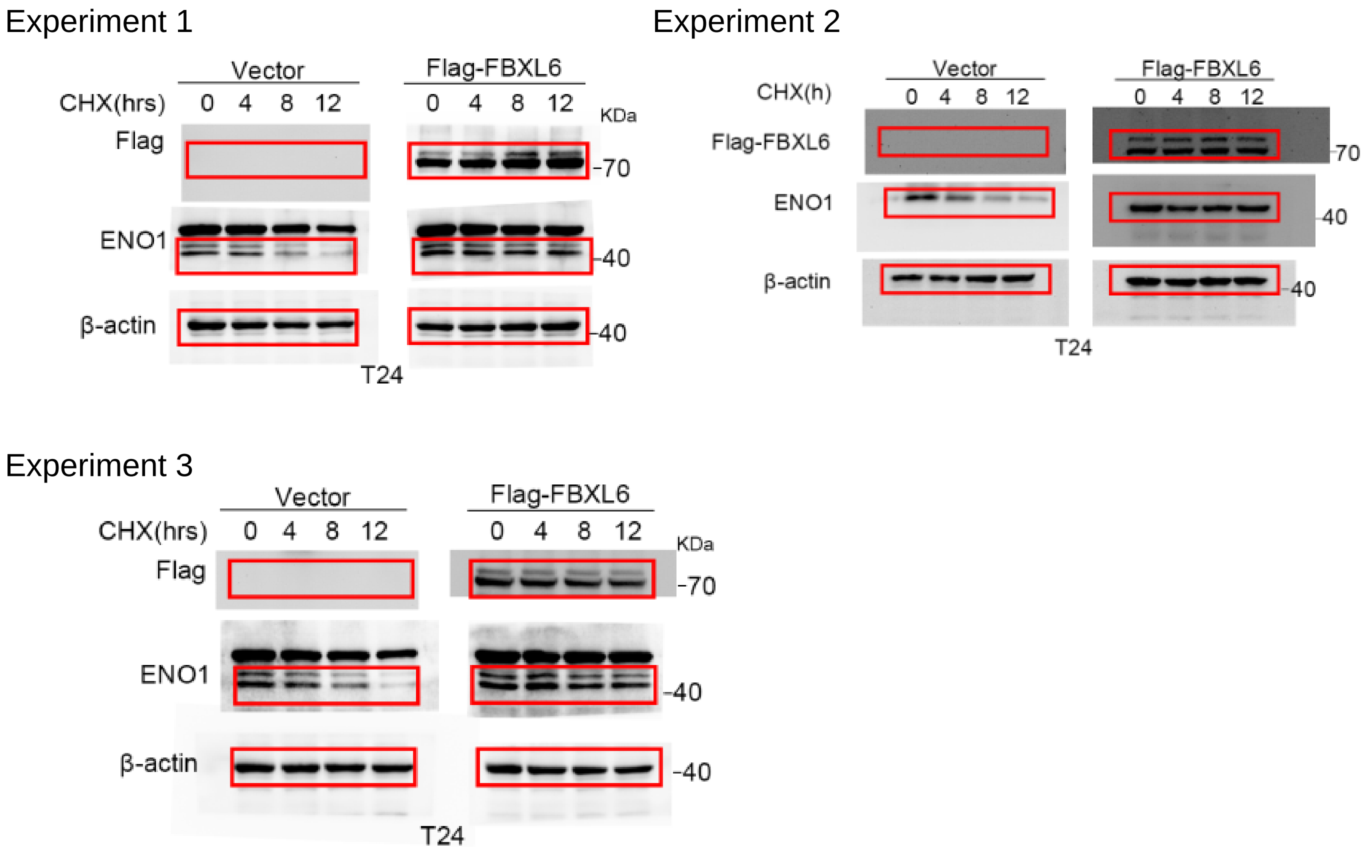

Supplementary Figure 5F

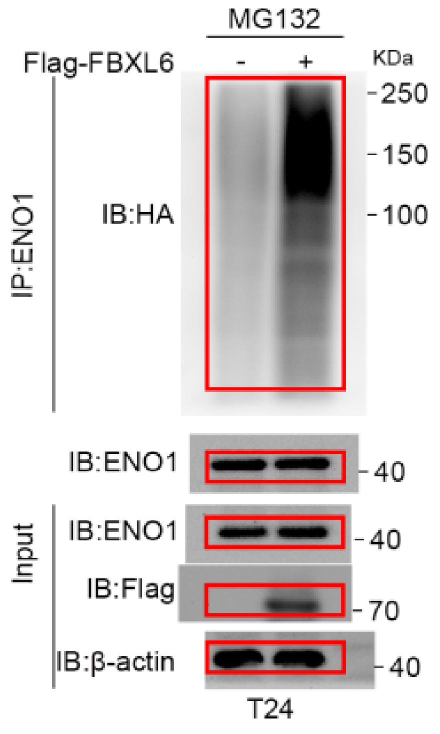

Supplementary Figure 5G

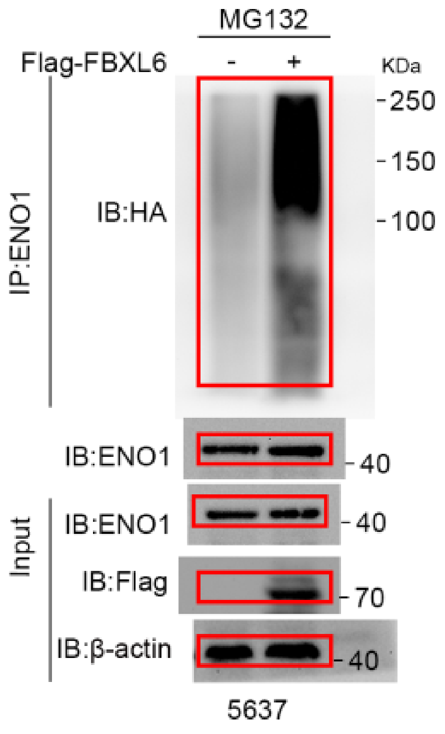

Supplement: Supplementary file 3 — Original Data of Western Blots [file 41420_2026_3130_MOESM3_ESM.pdf]
